# Testing whether the progression of Alzheimer’s disease changes with the year of publication, additional design, and geographical area: a modeling analysis of literature aggregate data

**DOI:** 10.1186/s13195-020-00630-5

**Published:** 2020-05-26

**Authors:** Ningyuan Zhang, Xijun Zheng, Hongxia Liu, Qingshan Zheng, Lujin Li

**Affiliations:** Center for Drug Clinical Research, Shanghai University of Traditional Chinese Medicine, No. 1200 Cailun Road, Shanghai, 201203 China

**Keywords:** Alzheimer’s disease, Disease progression model, Model-based meta-analysis, modeling and simulation

## Abstract

**Background:**

Our objectives were to develop a disease progression model for cognitive decline in Alzheimer’s disease (AD) and to determine whether disease progression of AD is related to the year of publication, add-on trial design, and geographical regions.

**Methods:**

Placebo-controlled randomized AD clinical trials were systemically searched in public databases. Longitudinal placebo response (mean change from baseline in the cognitive subscale of the Alzheimer’s Disease Assessment Scale [ADAS-cog]) and the corresponding demographic information were extracted to establish a disease progression model. Covariate screening and subgroup analyses were performed to identify potential factors affecting the disease progression rate.

**Results:**

A total of 134 publications (140 trials) were included in this model-based meta-analysis. The typical disease progression rate was 5.82 points per year. The baseline ADAS-cog score was included in the final model using an inverse U-type function. Age was found to be negatively correlated with disease progression rate. After correcting the baseline ADAS-cog score and the age effect, no significant difference in the disease progression rate was found between trials published before and after 2008 and between trials using an add-on design and those that did not use an add-on design. However, a significant difference was found among different trial regions. Trials in East Asian countries showed the slowest decline rate and the largest placebo effect.

**Conclusions:**

Our model successfully quantified AD disease progression by integrating baseline ADAS-cog score and age as important predictors. These factors and geographic location should be considered when optimizing future trial designs and conducting indirect comparisons of clinical outcomes.

## Background

Alzheimer’s disease (AD) is a degenerative neurological disease characterized by gradual memory loss and cognitive deterioration. Current FDA-approved AD drugs include cholinesterase inhibitors (CHEIs) and memantine. However, these drugs only provide symptomatic improvement and are unable to reverse cognitive decline in patients with AD [1–4]. Discovery of disease-modifying AD agents is urgent, yet none of the tested drugs showed statistical superior effects compared to placebos in large clinical trials [5–9]. From the perspective of new drug development, abnormal placebo decline might result in an increased risk of false-positive or false-negative outcomes. Therefore, to ensure a correct interpretation of the trial outcomes, it is necessary to predict or evaluate the cognitive decline in placebo-treated AD patients [7, 10].

The disease progression model, which describes the disease trajectory and underlying placebo effect, is widely used in AD clinical research to understand natural AD progression and inform future trial design [11, 12]. To date, several AD disease progression models for various outcomes have been published [11, 13–20]. However, as a large amount of new trial data has been published during the past decade, it is necessary to examine whether the disease progression rate has changed over such a long period. In addition, we noticed that subjects were usually allowed to continue their treatment of CHEIs or/and memantine as a background therapy in recent trials, which is a major distinction from the past trials [21–23]. Another major feature of AD clinical trials in recent years is their multinationality. Due to heterogeneities in cultures, access to healthcare, and prevalence of risk factors in different regions, the disease progression rate may differ between countries. However, to our knowledge, only one study has assessed AD progression across geographic regions [24]. They found that AD progression may differ across geographic regions; however, the heterogeneity across patient populations at baseline was not fully considered. Thus, these results need to be confirmed by more extensive data.

In this study, we re-retrieved AD trials and extracted ADAS-cog data from placebo groups to establish a summary-level AD disease progression model. The main objectives of our study were to (1) update the disease progression model, (2) investigate the potential correlation between disease progression and publication year, (3) investigate whether and to what extent disease progression differs between AD trials using or not using standard background therapy, and (4) explore the differences in disease progression in different geographic regions.

## Method

### Data collection

The public medical databases PubMed and Embase were searched for AD clinical trials on July 13, 2019. Key items were classified as “Alzheimer’s disease,” “clinical trial,” “placebo,” and “ADAS-cog.” Detailed information about the search strategy is provided in the supplementary materials.

Only clinical trials satisfying the following criteria were included in the analysis: (1) enrolled subjects must be diagnosed with dementia caused by AD, (2) trials must use parallel design and blind methods with placebo as the control group, (3) trials must report 11 items on the ADAS-cog scale as the primary or secondary outcome, (4) the sample size of the placebo group should be greater than ten, and (5) the language of the publication must be English.

The following information was extracted from the placebo group of the included AD studies to generate a database:

1. Characteristics of the publication and trial design, including title, main author, published year, clinical trial registration number, geographic region, trial length, randomized sample size, completed numbers, number of study sites, name of the test drug, whether subjects were allowed administration of standard background therapy (CHEIs or/and memantine) before entering and during the trial, and whether the study used add-on design.
2. Baseline demographic information such as stage of the disease, age, proportion of males, proportion of whites, proportion of patients with background therapy during the trial, proportion of APOE ε4 carriers, ADAS-cog score, Mini-Mental State Examination (MMSE) score, neuropsychiatric inventory (NPI) score, AD duration, and length of education.
3. Primary outcomes: mean change from baseline in the ADAS-cog score at each reported time point, the corresponding sample size and analyzed dataset (intention to treat, per protocol dataset, etc.), and imputation method of missing data (observed case OC or last observation carry forward LOCF). OC data were extracted prior to LOCF data if both were reported. GetData Graph Digitizer (version 2.26.0.20) was used to read longitudinal data if presented graphically.

### Disease progression model

#### Basic model

NONMEN 7.3 (Level 1.0, ICON Development Solutions, USA) was used to fit the time course of AD placebo response data. Model parameters were estimated by the first-order conditional estimation method with interaction (FOCE-I).

Placebo response, which consisted of both disease progression and the theoretical placebo effect, was defined as the mean change in ADAS-cog from baseline in the placebo group [11, 19, 25]. Disease progression refers to the natural change of cognitive function for AD subjects without any intervention, while the component of the placebo effect typically describes the temporary improvement due to the subjects’ expectations after entering the study. The structural model of the placebo response is shown in Eq. 1, where *α* is the slope parameter used to define the rate of linear disease progression and Pbo(*t*) is the time course of the placebo effect.

1 $$ S(t)=\alpha \times t+\mathrm{Pbo}(t) $$

The mathematical expression of the placebo effect models is shown in Eq. 2, where *β* represents the maximum extent of the placebo effect and ET_50_ is the time required to reach half of the maximum placebo effect. Besides, sigmoid *E*_max_, inverse Bateman, and an exponential model are also explored to describe the longitudinal placebo effect if possible.

2 $$ \mathrm{Pbo}(t)=\frac{\beta \times t}{{\mathrm{ET}}_{50}+t} $$

In AD trials, the disease status of cognitive function might either worsen or improve for different subjects. In addition, the nocebo effect may also affect cognitive function. Hence, inter-study variability for *α* and *β* was modeled using a proportional error model to allow individual parameter estimates to be either positive or negative (Eq. 3). For other structure model parameters, an exponential error model was used because their values were always positive (Eq. 4). In Eqs. 3 and 4, *P*_*i*_ is the individual parameter estimate of the *i*th study, and *P*_pop_ is the population parameter estimate. *η*_*i*_ is the random effect for the *i*th study to characterize the deviation between *P*_*i*_ and *P*_pop_, which was assumed to follow a normal distribution with a mean of 0 and variance of *ω*^2^. The residual error *ε* between the measured observation *Y* and the individual prediction *F* was modeled using an additive model and then weighted by the inverse of the square root of the corresponding sample size *N* (Eq. 5). *ε* was assumed to be normally distributed, with a mean of 0 and variance of *δ*^2^.

3 $$ {P}_i={P}_{\mathrm{pop}}\times \left(1+{\eta}_i\right) $$

4 $$ {P}_i={P}_{\mathrm{pop}}\times {e}^{\eta_i} $$

5 $$ Y=F+\frac{\varepsilon }{\sqrt{N}\ } $$

#### Covariate screening

The correlation between the inter-study variability of basic model parameters and the candidate covariates was explored after the establishment of the basic model. The dichotomous covariates, such as whether using an add-on design, were tested on the structure model parameters by introducing indication variables 0 and 1. Continuous covariates were tested using a linear equation or power function after normalization of the median value. In particular, the ceiling effect of the ADAS-cog scale should be noted when examining the baseline ADAS-cog effect on the rate of disease progression. When possible, the inverse U-type function was used to describe the nonlinear relationship between ADAS-cog effect and the rate of disease progression [13, 16], as shown in Eq. 6, where *α*_typical_ is the typical population estimate of *α*, ADAS_baseline_ is the baseline ADAS-cog score, ADAS_median_ is the median value among the included studies, and *θ* is the correction factor to control the shape of the curve.

6 $$ \alpha ={\alpha}_{\mathrm{typical}}\times {\left(\frac{{\mathrm{ADAS}}_{\mathrm{baseline}}}{{\mathrm{ADAS}}_{\mathrm{median}}}\times \frac{70-{\mathrm{ADAS}}_{\mathrm{baseline}}}{70-{\mathrm{ADAS}}_{\mathrm{median}}}\right)}^{\theta } $$

Covariate screening was performed in a stepwise manner. Evaluation of the covariate was based on the change value in the objective function value (OFV) using the likelihood ratio test method, decrease in inter-study variability, and prior knowledge of plausibility. In the forward inclusion step, a reduction in OFV of more than 3.84 points (*χ*^2^, *P* < 0.05, df = 1) was used as the criterion. Upon establishing the full model, the included covariates were removed successively to evaluate their necessity. An increase in OFV of more than 6.64 (*χ*^2^, *P* < 0.01, df = 1) was considered statistically significant to retain this covariate in the final model.

#### Model evaluation

Three methods were used to evaluate the model quality as follows [26]. (1) The diagnostic graphs were used to assess the consistency between the model prediction and the observation and inspect the randomness of the residual error distribution. (2) A non-parametric bootstrap method was used to generate 1000 new datasets by random sampling with replacement from the original dataset and using the identical model structure to fit and re-estimate the model parameters. The final model parameter estimates and their 95% confidence intervals (CI) were then compared with the median and 95% quantile of the bootstrap replicates to verify the precision and stability of the final model estimation. (3) Monte Carlo simulations were performed on 1000 template datasets based on the final model parameter estimates. Through a visual predictive check, the median and the 95% prediction interval of the simulated time course of ADAS-cog were plotted and overlapped with the observed data to qualify the predictive performance of the final model.

#### Subgroup analyses

We conducted subgroup analyses of disease progression rate (*α*) and maximum placebo effect (*β*) with regard to geographic regions, trial design (add-on design or not), and publication year (before or after 2008). The analysis method included two main steps. The first step was to obtain the estimated values of model parameters and their standard errors of each study [27]. The original individual estimates of model parameters were then adjusted by inverse calculation of covariate functions, which aimed to deduce the varying degrees of covariate impact across different studies [28, 29]. In the second step, the estimated model parameters were pooled under these predefined subgroups using a random effect meta-analysis of a single mean to obtain the overall mean and the 95% CI.

## Results

### Characteristics of the included studies

**Fig. 1:**
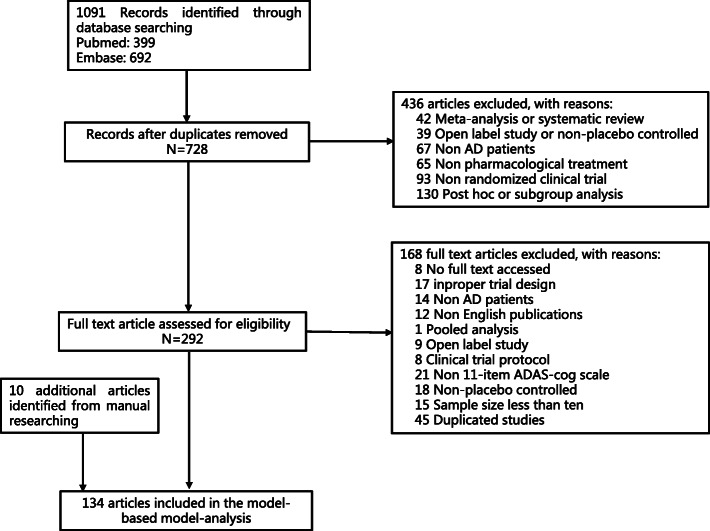
The flow diagram illustrating the inclusion and exclusion of studies into the final analysis

Finally, 134 articles, which involved 140 independent clinical trials and 19,210 placebo subjects, were eligible for inclusion. The screening flow chart and the reference list are shown in Fig. 1 and Additional file. The total number of ADAS-cog observations was 455. The median value (minimum–maximum) of baseline age, ADAS-cog score, and MMSE score were 73.5 years (58.0–81.7), 24.5 points (13.1–39.3), and 19.4 (13.2–25.4) points, respectively. The number of studies using the add-on design was 27, all of which were published after 2008 (Additional file Figure 1). The baseline demographic characteristics were similar as in those studies not using the add-on design. Baseline ADAS-cog scores for trials published after 2008 were significantly lower than those published before 2008 (− 2.56, 95% CI − 3.96, − 1.15). The other baseline demographics were comparable between the two groups (Table 1).

**Table 1:** Demographic characteristics of the included studies

	Overall	Trial with non-add-on design	Trial with add-on design	Trials before 2008	Trials after 2008
Number of trials	140	113	27	64	76
Total sample size	19,210	14,556	4654	7008	12,202
Sample size per arm*	102 (11, 746)	100 (11, 746)	132 (21, 479)	107 (14, 327)	101 (11, 746)
Age, years*	73.5 (58.0, 81.7)	73.3 (58.0, 81.7)	74.1 (68.0, 79.4)	73.2 (58.0, 78.9)	73.6 (64.6, 81.7)
Gender, male (%)*	42.1 (0, 98.0)	41.2 (0,67)	43.3 (0, 98.0)	42.6 (27.1, 67.0)	41.9 (0, 98.0)
Race, white (%)*	93.7 (0, 100)	93.1 (0, 100)	94.1 (0, 100)	92.6 (0, 100)	94.1 (0, 100)
APOE ε4 carrier, %*	58.4 (0, 100)	58.1 (0, 100)	58.3 (46.6, 74.0)	60.0 (53.0, 67.6)	57.9 (0, 100)
ADAS-cog 11, points*	24.5 (13.1, 39.3)	24.8 (13.1, 39.3)	24.1 (17.6, 28.0)	26.4 (16.2, 39.3)	24.0 (13.1, 32.2)
NPI score, points*	9.3 (5.0, 21.8)	9.6 (5.0, 21.1)	9.0 (5.8, 21.8)	11.0 (8.7, 16.1)	9.1 (5.0, 21.8)
MMSE score, points*	19.4 (13.2, 25.4)	19.4 (13.2 25.4)	19.5 (15.6, 22.2)	19.1 (13.2, 22.5)	20.1 (15.6, 25.4)
Publication year, no. (%)					
Before 2008	64 (45.7)	64 (61.2)	0 (0)	–	–
After 2008	76 (54.3)	49 (38.8)	27 (100)	–	–

*Reported mean data were express as median (minimum–maximum)

The countries where trials were conducted were combined into regions based on ethnicity and geography. Regions were as follows: North America (the USA and Canada), Europe/Oceania (the UK, France, Germany, Italy, the Netherlands, Norway, Poland, Russia, Ukraine, Spain, Sweden, Australia, and New Zealand), East Asia (China, Japan, and Korea), Middle Asia (Iran and Turkey), and South America (Brazil and Mexico). If the subjects of a study came from two or more of the above-listed regions, the trial was classified as multinational. The baseline information from the different geographic regions is summarized in Table 2. Most AD trials were conducted in Western countries, and their demographic characteristics were comparable to those of East Asian trials. The only exception was that more female patients were included in East Asian trials. Middle Asian trials enrolled a higher proportion of male patients with AD than other regions. The baseline ADAS-cog score was highest in the South American population, while a similar distribution was found among other regions.

**Table 2:** Characteristics of the included studies across different regions

	International	North America	Europe/Oceania	East Asia	Middle Asia	South America
Number of trials	56	56	15	5	5	3
Total sample size	10,842	6855	799	457	98	159
Sample size per arm*	135 (19, 639)	102 (11, 746)	40 (14, 153)	37 (19, 268)	20 (15, 25)	18 (12, 129)
Age, years*	73.2 (68.3, 78.3)	75.0 (58.0, 81.7)	72.1 (64.6, 79.8)	74.5 (69.0, 75.4)	73.1 (67.7, 73.7)	72.6 (71.7, 74.5)
Gender, male (%)*	40.3 (30, 59)	45.0 (0, 98)	38.0 (24.0, 53.3)	31.8 (23.8, 37.0)	52.1 (45, 60)	38.9 (27.1, 41.7)
APOE ε4 carrier, %*	58.0 (0, 72)	60.0 (41.3, 100)	51.1 (44.0, 58.8)	54.1**	N.A.	N.A.
ADAS-cog 11, points*	24.7 (18.9, 35.8)	23.4 (13.1, 32.2)	24.5 (15.4, 34.3)	26.9 (23.9, 33.5)	25.6 (17.1, 39.3)	36.7 (29.3, 39.0)
NPI score, points*	10.3 (6.2, 21.8)	8.65 (5.0, 16.1)	9.2 (7.1, 11.2)	9.1 (8.8, 9.3)	N.A.	N.A.
MMSE score, points*	19.4 (15.5, 22.9)	20.3 (17.0, 25.4)	19.3 (16.8, 23.6)	16.1 (14.6, 16.6)	13.2**	17.4 (17.2, 17.6)

*N.A* no available information was reported

*Reported mean data were express as median (minimum–maximum)

**Only one study reported the relevant information

Detailed demographic information of the included studies is listed in Additional file Table 1.

### Disease progression model

Most of the ADAS-cog data points included in the analysis were measured for 2 years, except for four observations derived from one study [30]. Only placebo data acquired during a period 2 years were modeled because long-term observations might have biased the estimation of disease progression rate. The results of the structural model exploration showed that the disease progression model combined with the *E*_max_ model to describe the placebo effect was the most appropriate basic model because of its lowest AIC value and higher precision of parameter estimations compared with those using the exponential or sigmoid *E*_max_ model. When using the inverse Bateman function, the offset rate constant of the placebo effect was difficult to estimate because the duration of some clinical trials included in this study was less than 12 weeks, and the minimization process for parameter estimation failed to converge successfully.

**Fig. 2:**
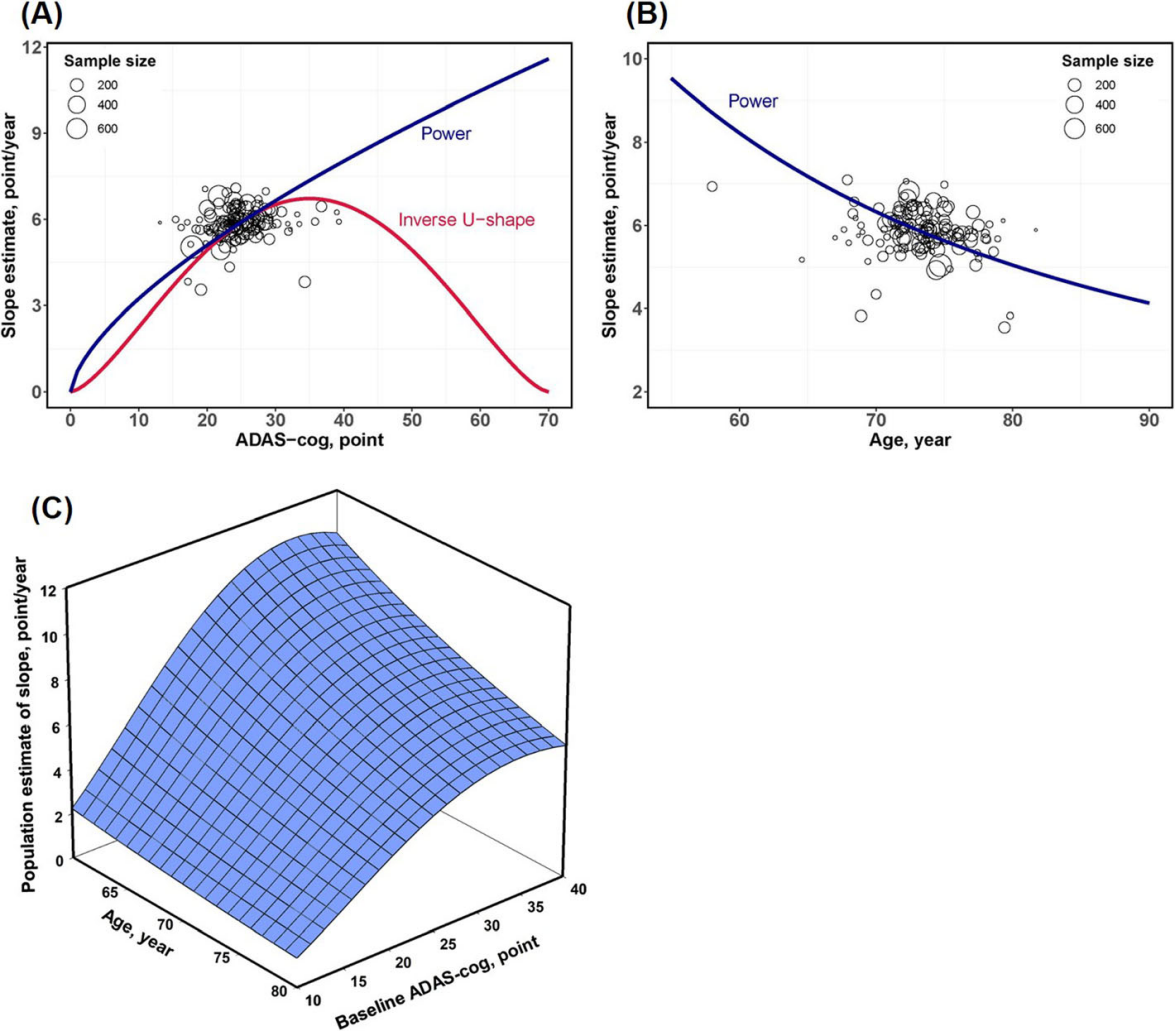
**a** Plot of the individual estimate of disease progression rate *α* derived from the base model versus the baseline ADAS-cog score. The two parameter-covariate relationships, power and U-type function, were demonstrated by using blue and red lines, respectively. **b** Plot of the individual estimate of disease progression rate *α* derived from the base model versus the baseline age. The blue fitted line demonstrated the correlation between them. In plot (**a**) and (**b**), each black circle stands for one independent study, and the circle size is proportional to the corresponding sample size. **c** A surface diagram to describe the relationship among age, ADAS-cog score, and the disease progression rate. Younger patients with higher baseline ADAS-cog score were predicted to manifest faster cognitive decline

The parameter estimates of the final model are shown in Table 3. Covariate screening analysis demonstrated that both baseline ADAS-cog score and age had a significant impact on the disease progression rate (*P* < 0.05). The relationship between the individual estimate of the disease progression rate from the basic model and the baseline ADAS-cog score showed an obvious nonlinear correlation, as shown in Fig. 2a. In this study, the inverse U-type model was superior to the power model for assessing the baseline ADAS-cog effect as the higher degree of OFV reduction compared with the basic model (10.884 versus 8.595), and the inverse U-type model was more consistent with the nonlinearity for boundary scale data, which is characterized by the faster disease progression rate in the moderate AD population than in the mild and severe AD populations. In addition, covariate analysis also found a negative correlation between age and disease progression rate (Fig. 2b). Younger patients with AD deteriorated faster in cognitive function than elderly AD patients. Compared with the basic model, the inter-study variability of disease progression rate decreased from 18.5 to 14.8% after the introduction of the effects of ADAS-cog and age as covariates. The final mathematical expression of the population estimate of *α* is shown in Eq. 7. For the AD population with an average age of 73.5 years and ADAS-cog baseline score of 24.5 points, the typical value of change rate in ADAS-cog scale was estimated to be 0.112 points/week (5.82 points/year). Theoretically, the disease progression rate was the fastest when the ADAS-cog score reached the inflection point of 35.5. The curved surface among the ADAS-cog baseline score, age, and population estimate of *α* is shown in Fig. 2c.

**Table 3:** Parameter estimates of the final disease progression model

Parameters	Estimates (RSE%)	NONMEN 95% CI	Bootstrap median	Bootstrap 95%PI
Fixed effect				
*α*, points/week	0.112 (6.5)	0.098 ~ 0.126	0.114	0.102 ~ 0.139
*β*, points	− 1.87 (26.4)	− 2.84 ~ − 0.902	− 2.03	− 4.28 ~ − 1.27
ET_50_, weeks	7.99 (50.4)	0.091 ~ 15.9	8.91	3.22 ~ 25.4
Covariate				
*θ*_baseline ADAS-cog score_	1.53 (34.8)	0.487 ~ 2.57	1.46	0.581 ~ 2.96
*θ*_baseline age_	− 2.17 (29.1)	− 3.41 ~ − 0.931	− 1.95	− 3.73 ~ − 0.625
Random effect				
*η*1 for *α*, %	14.8 (43.0)	2.33 ~ 27.3	15.5	2.9 ~ 27.3
*η*2 for *β*, %	72.5 (15.2)	50.9 ~ 94.1	74.4	53.3 ~ 106.6
Pearson correlation	− 0.717	NA	–	–
Residual error				
*σ*_addition_, points	5.64 (6.8)	4.89 ~ 6.39	5.54	4.83 ~ 6.26

7 $$ \alpha =0.112\times {\left(\frac{\mathrm{age}}{73.5}\right)}^{-2.17}\times {\left(\frac{{\mathrm{ADAS}}_{\mathrm{baseline}}}{24.5}\times \frac{70-{\mathrm{ADAS}}_{\mathrm{baseline}}}{45.5}\right)}^{1.53} $$

In the final model, the population estimate of *β* was − 1.87 points. The inter-study variability of *β* was estimated to be 72.5%, indicating that the placebo effect varied largely across trials. The sign of the individual estimate *β* was positive in only seven studies, which suggested that cognitive function deteriorated more in these studies and it was unclear whether this could be attributed to the nocebo effect. The estimated population value of ET_50_ was 7.99 weeks, and its inter-study variability was fixed at 0 because of the very large shrinkage value. Thus, it was anticipated that the plateau of the placebo effect might be reached at approximately 16 weeks in clinical trials. In addition, a significant negative correlation was found between the inter-study variabilities of *α* and *β*, and the corresponding Pearson correlation coefficient was − 0.717.

It was not found that add-on design and the proportion of subjects receiving background treatment during the trial and publication year had a significant impact on the disease progression rate. In addition, we did not find any factors that had a significant effect on the theoretical maximum placebo effect. Since information regarding the duration of education, NPI score, AD duration, proportion of APOE ε4 carriers, and proportion of whites were not reported in more than 30% of the studies, these variables were not included in the covariate screening step.

### Subgroup analyses

**Fig. 3:**
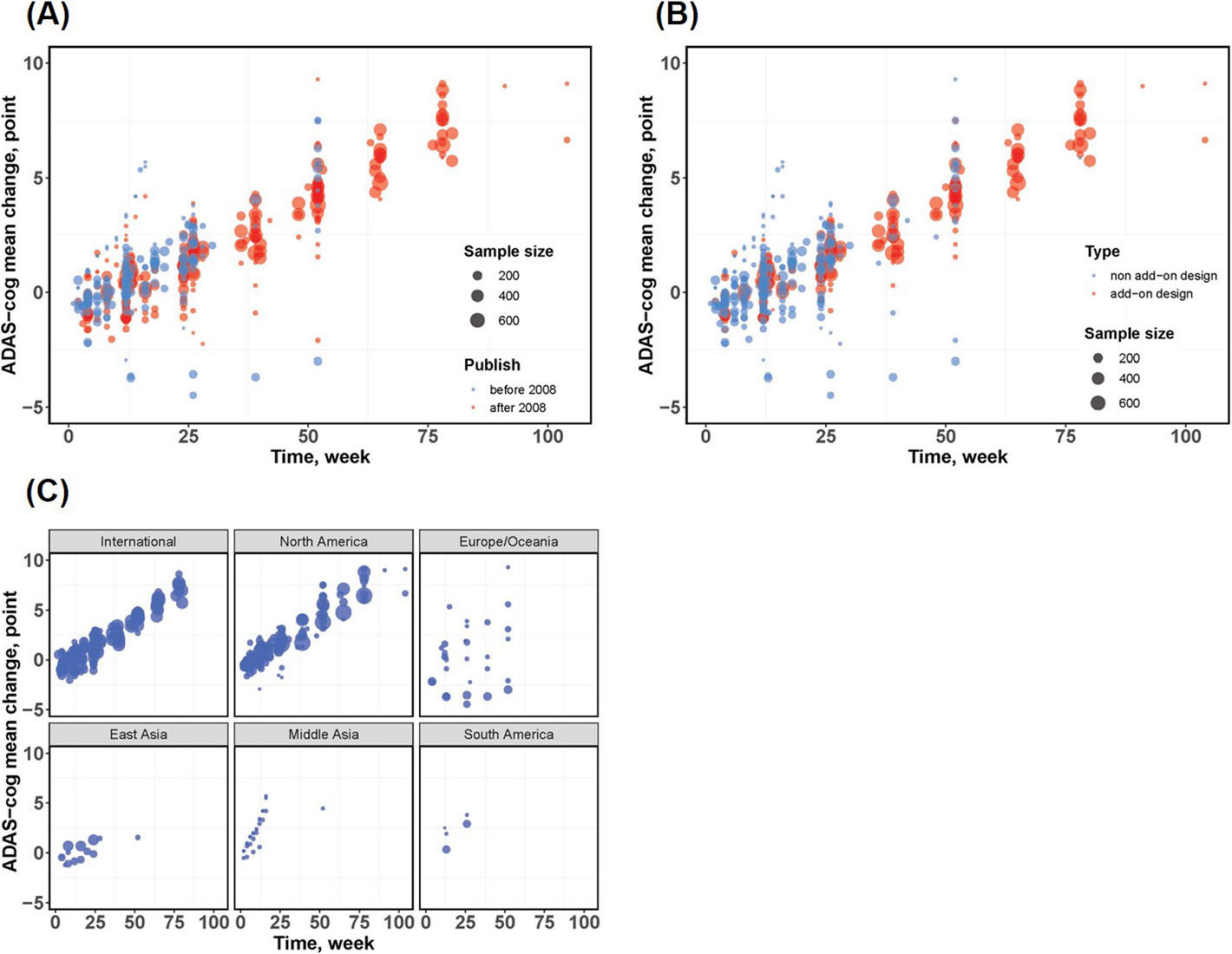
Observed mean change from baseline in ADAS-cog score under different stratification scenes; the point size is proportional to the corresponding sample size. **a** Published before 2008 versus after 2008; the red points and blue points stand for trials published after 2008 and before 2008, respectively. **b** Add-on design versus non-add-on design; the red points and blue points stand for add-on trials and trials not using add-on design, respectively. **c** Different geographic locations

The observed mean change from baseline in the ADAS-cog score under different stratifying factors is shown in Fig. 3. The results of the subgroup analysis of disease progression rate *α* and maximum placebo effect *β* are shown in Table 4. It was revealed that the disease progression rate published before 2008 was significantly higher than that published after 2008. However, after correcting for baseline ADAS-cog score and age effect, this significant difference disappeared (Table 4). This is why the publication year could not be introduced into the model as a covariate. Because the age of subjects was comparable between the trials published before or after 2008, we hypothesized that the decline rate of ADAS-cog score in the placebo group tended to decrease in recent AD trials, which might be driven by the relatively lower baseline ADAS-cog score. In addition, no difference was found between trials using the add-on design and those not using the add-on design.

**Table 4:** Subgroup analysis of disease progression rate

	Uncorrected disease progression rate, points/year, mean (95% CI)	Covariate-corrected disease progression rate, points/year, mean (95% CI)	Maximum extent of placebo effect, points mean (95% CI)
Overall	5.77 (5.60, 5.94)	5.86 (5.75, 5.97)	− 1.89 (− 2.08, − 1.69)
Publication year			
< 2008	6.13 (5.89, 6.39)	5.92 (5.80, 6.04)	− 1.70 (− 2.04, − 1.36)
≥ 2008	5.56 (5.40, 5.73)	5.81 (5.72, 5.92)	− 2.03 (− 2.24, − 1.81)
Trial design			
Add-on	5.53 (5.26, 5.80)	5.85 (5.69, 6.00)	− 1.92 (− 2.25, − 1.59)
Non-add-on	5.84 (5.64, 6.04)	5.87 (5.74, 6.01)	− 1.88 (− 2.11, − 1.65)
Regions			
International	5.81 (5.66, 5.97)	5.78 (5.67, 5.90)	− 2.17 (− 2.39, − 1.95)
North America	5.62 (5.35, 5.89)	6.11 (6.00, 6.23)	− 1.50 (− 1.69, − 1.31)
Europe/Oceania	5.58 (4.68, 6.48)	5.54 (4.79, 6.29)	− 2.35 (− 3.80, − 0.90)
East Asia	5.67 (5.15, 6.19)	5.45 (4.93, 5.97)	− 2.43 (− 3.45, − 1.41)
Middle Asia	7.00 (5.98, 8.02)	6.76 (6.20, 7.31)	− 0.01 (− 1.31, 1.28)
South America	7.27 (6.49, 8.06)	6.27 (5.48, 7.06)	− 1.35 (− 2.11, − 0.60)

The values have been converted to annual disease progression rate, *α* (points/week) × 52 weeks/year

**Fig. 4:**
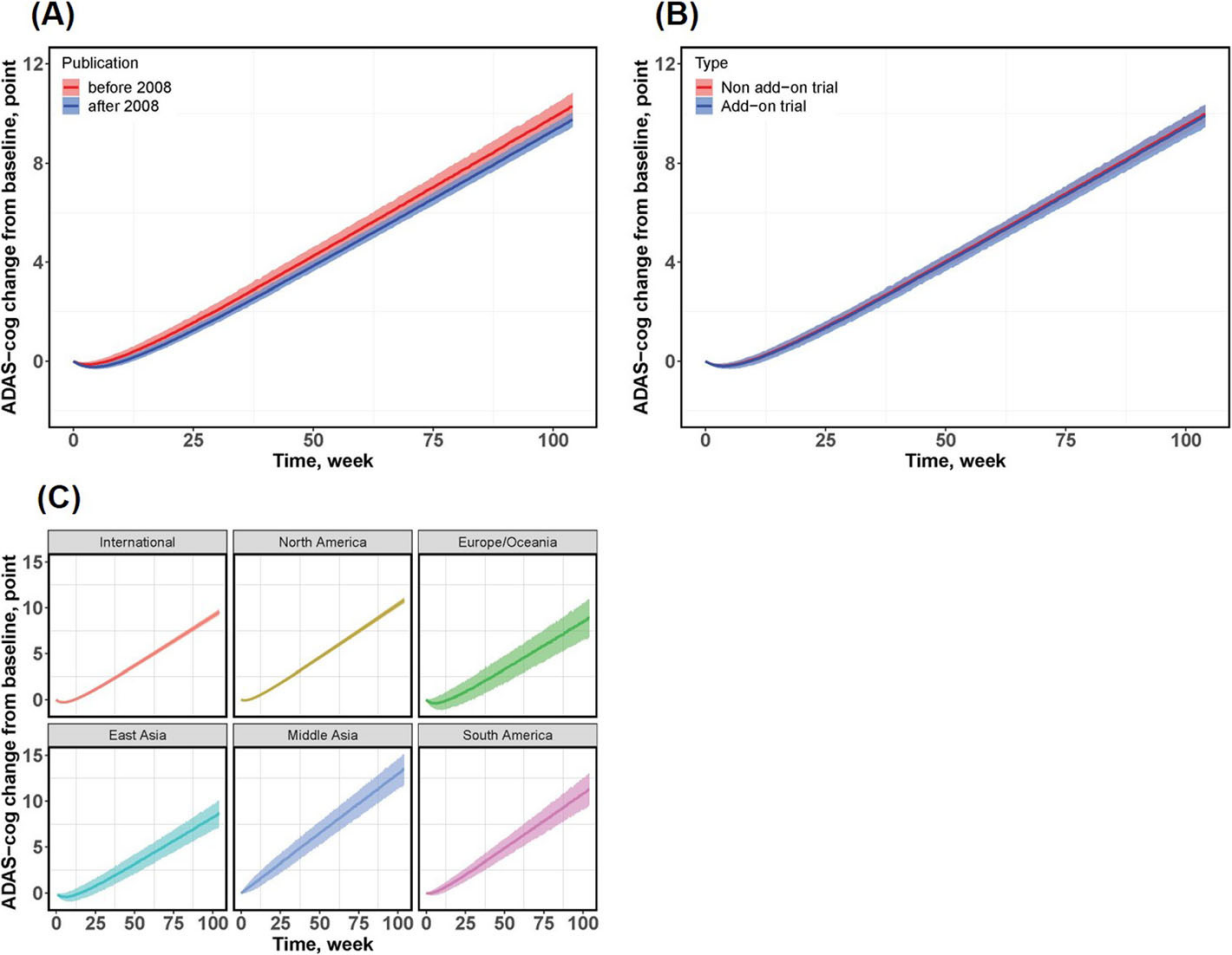
Typical time course of the change of ADAS-cog score from baseline under different stratification scenes. **a** Published before 2008 versus after 2008. **b** Add-on design versus non-add-on design. **c** Different geographic locations. The shadows are the model-predicted 95% CI of the placebo response. The solid line is the typical value of the model-predicted placebo response

An interesting finding was that disease progression varied across geographic regions. Even after correcting for the baseline ADAS-cog score and age effect, trials conducted in Middle Asian countries had a higher disease progression rate than trials conducted in international and East Asian countries. East Asian trials showed the slowest disease progression rate compared to other trials. The theoretical maximum placebo effect in international multiple trials was significantly higher than that in North American populations, while the Middle Asian population showed the weakest placebo effect than any other region. Based on the model parameters obtained by subgroup analyses, we simulated the typical time course of change in ADAS-cog score from baseline under different stratification factors (Fig. 4).

### Model evaluation and validation

The median values of the model parameters obtained from the non-parametric bootstrap analysis were consistent with the parameter estimates of the final model. In addition, the 95% CI of the covariate correction factors did not contain 0, further verifying the reliability of the covariate analysis results.

The quality of the fit of the final model was relatively good (Additional file Figure 2). Both population predictions and individual predictions correlated well with the observations. The model predictions were evenly distributed on both sides of the standard line without obvious deviation. The absolute values of the conditional weighted residuals were less than 6 and no systematic trend was found over time and the population predictions. The visual predictive check (Additional file Figure 3) showed that the median and the 2.5% and 97.5% quantiles of the observations fell within the 95% CI of the quantile predicted by the final model, indicating that the final model could reflect the central trend and variability of the observation values. Thus, the final model had good reproducibility for the original data and good predictive capabilities.

## Discussion

This model-based meta-analysis integrated longitudinal data of ADAS-cog in the placebo group from published AD clinical trials from 1992 to 2019, extended the observation period to 2 years, and reconstructed the literature-level AD disease progression model. We found that baseline ADAS-cog score and age were two important factors influencing the disease progression rate, consistent with the results of individual-level studies based on the ADNI database [16, 31].

Previous studies based on individual data have found that the relationship between disease progression rate and baseline ADAS-cog score is nonlinear [32, 33]. Therefore, we used the inverse U-type model in this study to describe the nonlinear relationship between disease progression rate and ADAS-cog score. However, since the maximum baseline ADAS-cog score of the subjects included in this study at the summary level was no more than 40 points, the trend that the disease progression rate decreases with the increase in the baseline ADAS-cog score was not obvious. Thus, it is necessary to be careful to extrapolate the disease progression rate when the baseline ADAS-cog score is greater than 40 points at the summary level. Compared with the study by Ito et al., our study found that baseline age also had a significant impact on the disease progression rate. The estimated correction coefficient for the age effect was − 2.17, which was slightly lower than the value of − 1.8 obtained by using individual data modeling [13]. The cognitive decline in the younger AD population was faster than that of the elderly AD population. For example, the disease progression rate of the 60-year-old AD population was estimated to be about 1.87 times as fast as that of the 80-year-old AD population. Based on the sample size, baseline ADAS-cog score, and age, the final model could predict the time course of ADAS-cog in the placebo group as a historical control in future AD trials, whereas model predictions were compared with observations from clinical trials to aid in the evaluation and interpretation of trial outcomes [25, 34, 35].

The guidelines for AD published around 2008 recommend CHEIs and memantine as the standard treatment for AD patients [1, 2]. The number of AD trials designed with these drugs as an add-on treatment has increased since then. In this study, we found that the disease progression rate was almost the same between trials with add-on design and non-add-on design. This is consistent with the conclusion that currently approved drugs do not change the disease progression rate. The enrolled subjects were required to have been treated with these drugs at a stable dose for at least 1 to 3 months before entering the add-on trials. The drug efficacy was nearly close to the plateau at the beginning of the trial, and it was expected not to affect the estimation of the placebo response.

In this study, a subgroup analysis of the disease progression rate according to the year of publication was conducted to test the hypothesis that clinical care and patient management may improve over time. We found that the mean annual increase in ADAS-cog score in AD trials published after 2008 was significantly lower than that before 2008. However, when the baseline ADAS-cog score was corrected, the impact of the publication year on the disease progression rate disappeared, which suggested that the publication effect was due to the fact that more patients with mild AD were included in recent trials. The above-described results suggest that when performing a systematic review or meta-analysis, the data of add-on trials and non-add-on trials as well as the data published in the past 10 years and data published more than 10 years ago can be combined and analyzed to increase the richness of the available data.

Clinical trials of AD have been conducted globally, and we found that clinical trials in Middle Asian countries showed rapid disease progression in the placebo group (Table 4). It should be noted that the sample size of trials in Middle Asia was relatively small, which might have resulted in a biased estimation of the placebo decline rate. Meanwhile, by comparing the original and adjusted disease progression rates, we concluded that the higher decline rate in South American trials was due to the inclusion of patients with more severe AD. Importantly, we found that the covariate-adjusted disease progression rate in East Asia was significantly lower than that in North America. This conclusion is consistent with a previous study [24], in which, however, the baselines of subjects in different regions were not comparable and the conclusion was questioned. Cerebrovascular insufficiency was associated with the onset and progression of cognitive dysfunction [36], and we suspected vascular load difference between western and eastern populations might contribute to the relatively slower decline rate in East Asian countries. Final model simulation suggested that the ADAS-cog change of East Asian populations was flatter than that of North American populations. The typical change scores from baseline in ADAS-cog of East Asian and North American populations at 6 months were 0.9 (95% CI 0.03–1.68) and 1.91 (95% CI 1.75–2.06), respectively. Therefore, differences in disease progression and placebo effects from different regions, especially in East Asia and North America, need to be noted when conducting multicenter clinical trials. However, it should be noted that most of the ADAS-cog data points in East Asia included in the analysis were measured in 6 months, and the trajectory of disease progression in East Asia over a longer treatment duration still requires further data support.

This study has some limitations. First, we did not examine the effects of some important covariates on disease progression rate. For example, there is substantial evidence that APOE ε4 alleles are a high-risk factor for AD progression [15]. Other important biomarkers, such as the ratio of p-tau_181P_ over Aβ_1–42_ in the cerebrospinal fluid and hippocampal volume [15, 17, 18], were also not possible to account for because the proportion of studies that did not include these key covariates was too high to be integrated into the model. Second, the extrapolation of the final model to predict ADAS-cog changes after 2 years might be limited. There were some deviations between model predictions and observations when predicting long-term placebo response in mild-to-moderate AD subjects (Additional file Figure 4). Finally, the time lag between the recruitment of AD patients into clinical trials and the published year might interfere with drawing the conclusion of the publication year effect [37].

## Conclusions

To our knowledge, this study includes the highest number of clinical trials so far in the field of AD disease progression modeling. We found that the baseline ADAS-cog score and age are two important predictors for the disease progression rate in AD. The disease progression of AD is not affected by publication year and add-on design but shows different regional differences. The disease progression rate in East Asia is significantly lower than that in North America. These conclusions should be fully considered when designing future AD trials or conducting indirect comparisons of clinical outcomes.

## Supplementary information

**Additional file 1.** Supplementary materials, tables, and figures.

## Data Availability

The datasets used and/or analyzed during the current study are available from the corresponding authors on reasonable request.

## Abbreviations

AD: Alzheimer’s disease
ADAS-cog: Cognitive subscale of the Alzheimer’s Disease Assessment Scale
aMCI: Amnestic mild cognitive impairment
CHEIs: Cholinesterase inhibitors
CI: Confidence interval
MMSE: Mini-Mental State Examination
NPI: Neuropsychiatric inventory
VPC: Visual predictive check
