# Supplementary material for: Testing whether the progression of Alzheimer’s disease changes with the year of publication, additional design, and geographical area: a modeling analysis of literature aggregate data
Source: Alzheimers Res Ther. 2020 May 26;12:64. doi: 10.1186/s13195-020-00630-5 (PMC7251914; doi:10.1186/s13195-020-00630-5)
Supplement: Supplementary file 1 — Additional file 1. Supplementary materials, tables, and figures. [file 13195_2020_630_MOESM1_ESM.docx]

**Supplementary materials**

**Search strategy**

The search key words were: (Ⅰ) alzheimer‘s disease OR alzheimer* OR AD OR senile dementia OR mild cognitive impairment OR MCI (Ⅱ) placebo control OR placebo OR double blind OR single blind OR blinded OR control group OR randomized OR random* OR randomized controlled trial OR controlled clinical trial (Ⅲ) ADAS-cog* OR Alzheimer* Disease Assessment Scale-cognitive subscale

The logical word “AND” was utilized to combine the three keyword groups. Otherwise, the type of article was limited to clinical trial or clinical trials/human. Upon completion of literature retrieval and screening, the references cited in relevant AD systematic reviews and meta-analyses were manually checked to ensure no omissions of important published studies.

**Articles included in the model-based meta-analysis**

[1] Vila-Castelar C, Ly JJ, Kaplan L, Van Dyk K, Berger JT, Macina LO, Stewart JL, Foldi NS (2019) Attention Measures of Accuracy, Variability, and Fatigue Detect Early Response to Donepezil in Alzheimer's Disease: A Randomized, Double-blind, Placebo-Controlled Pilot Trial. *Archives of clinical neuropsychology : the official journal of the National Academy of Neuropsychologists* **34**, 277-289.

[2] Bernard K, Gouttefangeas S, Bretin S, Galtier S, Robert P, Holthoff-Detto V, Cummings J, Pueyo M (2019) A 24-week double-blind placebo-controlled study of the efficacy and safety of the AMPA modulator S47445 in patients with mild to moderate Alzheimer's disease and depressive symptoms. *Alzheimer's and Dementia: Translational Research and Clinical Interventions* **5**, 231-240.

[3] Zhu CW, Grossman H, Neugroschl J, Parker S, Burden A, Luo X, Sano M (2018) A randomized, double-blind, placebo-controlled trial of resveratrol with glucose and malate (RGM) to slow the progression of Alzheimer's disease: A pilot study. *Alzheimer's and Dementia: Translational Research and Clinical Interventions* **4**, 609-616.

[4] Wang GH, Wang LH, Wang C, Qin LH (2018) Spore powder of ganoderma lucidum for the treatment of Alzheimer disease a pilot study. *Medicine (United States)* **97**.

[5] Voss T, Li J, Cummings J, Farlow M, Assaid C, Froman S, Leibensperger H, Snow-Adami L, McMahon KB, Egan M, Michelson D (2018) Randomized, controlled, proof-of-concept trial of MK-7622 in Alzheimer's disease. *Alzheimer's and Dementia: Translational Research and Clinical Interventions* **4**, 173-181.

[6] Rafii MS, Tuszynski MH, Thomas RG, Barba D, Brewer JB, Rissman RA, Siffert J, Aisen PS, Mintzer J, Lerner A, Levey A, Burke J, Sano M, Turner S, Zamrini E, Grill J, Marson D (2018) Adeno-associated viral vector (serotype 2)-nerve growth factor for patients with Alzheimer disease a randomized clinical trial. *JAMA Neurology* **75**, 834-841.

[7] Fullerton T, Binneman B, David W, Delnomdedieu M, Kupiec J, Lockwood P, Mancuso J, Miceli J, Bell J (2018) A Phase 2 clinical trial of PF-05212377 (SAM-760) in subjects with mild to moderate Alzheimer's disease with existing neuropsychiatric symptoms on a stable daily dose of donepezil. *Alzheimer's Research and Therapy* **10**.

[8] Egan MF, Kost J, Tariot PN, Aisen PS, Cummings JL, Vellas B, Sur C, Mukai Y, Voss T, Furtek C, Mahoney E, Mozley LH, Vandenberghe R, Mo Y, Michelson D (2018) Randomized trial of verubecestat for mild-to-moderate Alzheimer's disease. *New England Journal of Medicine* **378**, 1691-1703.

[9] Atri A, Frolich L, Ballard C, Tariot PN, Molinuevo JL, Boneva N, Windfeld K, Raket LL, Cummings JL (2018) Effect of Idalopirdine as Adjunct to Cholinesterase Inhibitors on Change in Cognition in Patients With Alzheimer Disease: Three Randomized Clinical Trials. *Jama* **319**, 130-142.

[10] NR R, RG T, RA R, JB B, MS R, CH vD, CR J, M S, DS K, R R, P S, DM G, S F, PS A (2017) A phase 3 trial of IV immunoglobulin for Alzheimer disease. *Neurology* **88**, 1768-1775.

[11] Nave S, Doody RS, Boada M, Grimmer T, Savola JM, Delmar P, Pauly-Evers M, Nikolcheva T, Czech C, Borroni E, Ricci B, Dukart J, Mannino M, Carey T, Moran E, Gilaberte I, Muelhardt NM, Gerlach I, Santarelli L, Ostrowitzki S, Fontoura P (2017) Sembragiline in Moderate Alzheimer's Disease: Results of a Randomized, Double-Blind, Placebo-Controlled Phase II Trial (MAyflOwer RoAD). *J Alzheimers Dis* **58**, 1217-1228.

[12] Gauthier S, Feldman HH, Schneider LS, Wilcock GK, Frisoni GB, Hardlund JH, Moebius HJ, Bentham P, Kook KA, Wischik DJ, Schelter BO, Davis CS, Staff RT, Bracoud L, Shamsi K, Storey JMD, Harrington CR, Wischik CM (2016) Efficacy and safety of tau-aggregation inhibitor therapy in patients with mild or moderate Alzheimer's disease: a randomised, controlled, double-blind, parallel-arm, phase 3 trial. *The Lancet* **388**, 2873-2884.

[13] Gault LM, Lenz RA, Ritchie CW, Meier A, Othman AA, Tang Q, Berry S, Pritchett Y, Robieson WZ (2016) ABT-126 monotherapy in mild-to-moderate Alzheimer's dementia: Randomized double-blind, placebo and active controlled adaptive trial and open-label extension. *Alzheimer's Research and Therapy* **8**.

[14] Florian H, Meier A, Gauthier S, Lipschitz S, Lin Y, Tang Q, Othman AA, Robieson WZ, Gault LM (2016) Efficacy and safety of ABT-126 in subjects with mild-to-moderate Alzheimer's disease on stable doses of acetylcholinesterase inhibitors: A randomized, double-blind, placebo-controlled study. *Journal of Alzheimer's Disease* **51**, 1237-1247.

[15] Choe YM, Kim KW, Jhoo JH, Ryu SH, Seo EH, Sohn BK, Byun MS, Bak JH, Lee JM, Yun HJ, Han MI, Woo JI, Lee DY (2016) Multicenter, randomized, placebo-controlled, double-blind clinical trial of escitalopram on the progression-delaying effects in Alzheimer’s disease. *International Journal of Geriatric Psychiatry* **31**, 731-739.

[16] Tajadini H, Saifadini R, Choopani R, Mehrabani M, Kamalinejad M, Haghdoost AA (2015) Herbal medicine Davaie Loban in mild to moderate Alzheimer's disease: A 12-week randomized double-blind placebo-controlled clinical trial. *Complementary Therapies in Medicine* **23**, 767-772.

[17] Maher-Edwards G, Watson C, Ascher J, Barnett C, Boswell D, Davies J, Fernandez M, Kurz A, Zanetti O, Safirstein B, Schronen JP, Zvartau-Hind M, Gold M (2015) Two randomized controlled trials of SB742457 in mild-to-moderate Alzheimer's disease. *Alzheimer's and Dementia: Translational Research and Clinical Interventions* **1**, 23-36.

[18] Lenz RA, Pritchett YL, Berry SM, Llano DA, Han S, Berry DA, Sadowsky CH, Abi-Saab WM, Saltarelli MD (2015) Adaptive, Dose-finding Phase 2 Trial Evaluating the Safety and Efficacy of ABT-089 in Mild to Moderate Alzheimer Disease. *Alzheimer Disease and Associated Disorders* **29**, 192-199.

[19] Henderson VW, Ala T, Sainani KL, Bernstein AL, Stephenson BS, Rosen AC, Farlow MR (2015) Raloxifene for women with Alzheimer disease: A randomized controlled pilot trial. *Neurology* **85**, 1937-1944.

[20] Gauthier S, Rountree S, Finn B, LaPlante B, Weber E, Oltersdorf T (2015) Effects of the Acetylcholine Release Agent ST101 with Donepezil in Alzheimer's Disease: A Randomized Phase 2 Study. *Journal of Alzheimer's Disease* **48**, 473-481.

[21] Gault LM, Ritchie CW, Robieson WZ, Pritchett Y, Othman AA, Lenz RA (2015) A phase 2 randomized, controlled trial of the α7 agonist ABT-126 in mild-to-moderate Alzheimer's dementia. *Alzheimer's and Dementia: Translational Research and Clinical Interventions* **1**, 81-90.

[22] Bowen RL, Perry G, Xiong C, Smith MA, Atwood CS (2015) A clinical study of lupron depot in the treatment of women with Alzheimer's disease: Preservation of cognitive function in patients taking an acetylcholinesterase inhibitor and treated with high dose lupron over 48 weeks. *Journal of Alzheimer's Disease* **44**, 549-560.

[23] Wilkinson D, Windfeld K, Colding-Jorgensen E (2014) Safety and efficacy of idalopirdine, a 5-HT6 receptor antagonist, in patients with moderate Alzheimer's disease (LADDER): a randomised, double-blind, placebo-controlled phase 2 trial. *Lancet Neurol* **13**, 1092-1099.

[24] Shinto L, Quinn J, Montine T, Dodge HH, Woodward W, Baldauf-Wagner S, Waichunas D, Bumgarner L, Bourdette D, Silbert L, Kaye J (2014) A randomized placebo-controlled pilot trial of omega-3 fatty acids and alpha lipoic acid in Alzheimer's disease. *Journal of Alzheimer's Disease* **38**, 111-120.

[25] Schwam EM, Nicholas T, Chew R, Billing CB, Davidson W, Ambrose D, Altstiel LD (2014) A multicenter, double-blind, placebo-controlled trial of the PDE9A inhibitor, PF-04447943, in Alzheimer's disease. *Curr Alzheimer Res* **11**, 413-421.

[26] Salloway S, Sperling R, Fox NC, Blennow K, Klunk W, Raskind M, Sabbagh M, Honig LS, Porsteinsson AP, Ferris S, Reichert M, Ketter N, Nejadnik B, Guenzler V, Miloslavsky M, Wang D, Lu Y, Lull J, Tudor IC, Liu E, Grundman M, Yuen E, Black R, Brashear HR (2014) Two phase 3 trials of Bapineuzumab in mild-to-moderate Alzheimer's disease. *New England Journal of Medicine* **370**, 322-333.

[27] Marek GJ, Katz DA, Meier A, Greco N, Zhang W, Liu W, Lenz RA (2014) Efficacy and safety evaluation of HSD-1 inhibitor ABT-384 in Alzheimer's disease. *Alzheimer's and Dementia* **10**, S364-S373.

[28] Haig GM, Pritchett Y, Meier A, Othman AA, Hall C, Gault LM, Lenz RA (2014) A randomized study of H3 antagonist ABT-288 in mild-to-moderate Alzheimer's dementia. *Journal of Alzheimer's Disease* **42**, 959-971.

[29] Grove RA, Harrington CM, Mahler A, Beresford I, Maruff P, Lowy MT, Nicholls AP, Boardley RL, Berges AC, Nathan PJ, Horrigan JP (2014) A Randomized, Double-Blind, Placebo-Controlled, 16-Week Study of the H3 Receptor Antagonist, GSK239512 as a Monotherapy in Subjects with Mild-to-Moderate Alzheimer's Disease. *Current Alzheimer Research* **11**, 47-58.

[30] Grimaldi LM, Zappala G, Iemolo F, Castellano AE, Ruggieri S, Bruno G, Paolillo A (2014) A pilot study on the use of interferon beta-1a in early Alzheimer's disease subjects. *J Neuroinflammation* **11**, 30.

[31] Galasko D, Bell J, Mancuso JY, Kupiec JW, Sabbagh MN, van Dyck C, Thomas RG, Aisen PS (2014) Clinical trial of an inhibitor of RAGE-Abeta interactions in Alzheimer disease. *Neurology* **82**, 1536-1542.

[32] Dysken MW, Sano M, Asthana S, Vertrees JE, Pallaki M, Llorente M, Love S, Schellenberg GD, McCarten JR, Malphurs J, Prieto S, Chen P, Loreck DJ, Trapp G, Bakshi RS, Mintzer JE, Heidebrink JL, Vidal-Cardona A, Arroyo LM, Cruz AR, Zachariah S, Kowall NW, Chopra MP, Craft S, Thielke S, Turvey CL, Woodman C, Monnell KA, Gordon K, Tomaska J, Segal Y, Peduzzi PN, Guarino PD (2014) Effect of vitamin E and memantine on functional decline in Alzheimer disease: the TEAM-AD VA cooperative randomized trial. *Jama* **311**, 33-44.

[33] Doody RS, Thomas RG, Farlow M, Iwatsubo T, Vellas B, Joffe S, Kieburtz K, Raman R, Sun X, Aisen PS, Siemers E, Liu-Seifert H, Mohs R (2014) Phase 3 trials of solanezumab for mild-to-moderate alzheimer's disease. *New England Journal of Medicine* **370**, 311-321.

[34] Shah RC, Kamphuis PJ, Leurgans S, Swinkels SH, Sadowsky CH, Bongers A, Rappaport SA, Quinn JF, Wieggers RL, Scheltens P, Bennett DA (2013) The S-Connect study: Results from a randomized, controlled trial of Souvenaid in mild-to-moderate Alzheimer's disease. *Alzheimer's Research and Therapy* **5**.

[35] Molloy DW, Standish TI, Zhou Q, Guyatt G (2013) A multicenter, blinded, randomized, factorial controlled trial of doxycycline and rifampin for treatment of Alzheimer's disease: The DARAD trial. *International Journal of Geriatric Psychiatry* **28**, 463-470.

[36] Doody RS, Raman R, Farlow M, Iwatsubo T, Vellas B, Joffe S, Kieburtz K, He F, Sun X, Thomas RG, Aisen PS, Siemers E, Sethuraman G, Mohs R (2013) A phase 3 trial of semagacestat for treatment of Alzheimer's disease. *New England Journal of Medicine* **369**, 341-350.

[37] Egan M, Yaari R, Liu L, Ryan M, Peng Y, Lines C, Michelson D (2012) Pilot randomized controlled study of a histamine receptor inverse agonist in the symptomatic treatment of AD. *Current Alzheimer Research* **9**, 481-490.

[38] Coric V, Van Dyck CH, Salloway S, Andreasen N, Brody M, Richter RW, Soininen H, Thein S, Shiovitz T, Pilcher G, Colby S, Rollin L, Dockens R, Pachai C, Portelius E, Andreasson U, Blennow K, Soares H, Albright C, Feldman HH, Berman RM (2012) Safety and tolerability of the γ-secretase inhibitor avagacestat in a phase 2 study of mild to moderate Alzheimer disease. *Archives of Neurology* **69**, 1430-1440.

[39] Andersen F, Viitanen M, Halvorsen DS, Straume B, Wilsgaard T, Engstad TA (2012) The effect of stimulation therapy and donepezil on cognitive function in Alzheimer's disease. A community based RCT with a two-by-two factorial design. *BMC Neurology* **12**.

[40] Vellas B, Sol O, Snyder PJ, Ousset PJ, Haddad R, Maurin M, Lemarié JC, Désiré L, Pando MP (2011) EHT0202 in Alzheimer's disease: A 3-Month, randomized, placebo-controlled, Double-Blind study. *Current Alzheimer Research* **8**, 203-212.

[41] Sano M, Bell KL, Galasko D, Galvin JE, Thomas RG, Van Dyck CH, Aisen PS (2011) A randomized, double-blind, placebo-controlled trial of simvastatin to treat Alzheimer disease. *Neurology* **77**, 556-563.

[42] Rafii MS, Walsh S, Little JT, Behan K, Reynolds B, Ward C, Jin S, Thomas R, Aisen PS (2011) A phase II trial of huperzine A in mild to moderate Alzheimer disease. *Neurology* **76**, 1389-1394.

[43] Nakamura Y, Imai Y, Shigeta M, Graf A, Shirahase T, Kim H, Fujii A, Mori J, Homma A (2011) A 24-Week, Randomized, Double-Blind, Placebo-Controlled Study to Evaluate the Efficacy, Safety and Tolerability of the Rivastigmine Patch in Japanese Patients with Alzheimeru2019s Disease. *Dementia & Geriatric Cognitive Disorders Extra* **1**, 163-179.

[44] Maher-Edwards G, Dixon R, Hunter J, Gold M, Hopton G, Jacobs G, Hunter J, Williams P (2011) SB-742457 and donepezil in Alzheimer disease: A randomized, placebo-controlled study. *International Journal of Geriatric Psychiatry* **26**, 536-544.

[45] Harrington C, Sawchak S, Chiang C, Davies J, Donovan C, Saunders AM, Irizarry M, Jeter B, Zvartau-Hind M, van Dyck CH, Gold M (2011) Rosiglitazone does not improve cognition or global function when used as adjunctive therapy to AChE inhibitors in mild-to-moderate alzheimer's disease: Two phase 3 studies. *Current Alzheimer Research* **8**, 592-606.

[46] Frölich L, Ashwood T, Nilsson J, Eckerwall G (2011) Effects of AZD3480 on cognition in patients with mild-to-moderate alzheimer's disease: A phase IIb dose-finding study. *Journal of Alzheimer's Disease* **24**, 363-374.

[47] DS G, T F, MJ M, G L (2011) A randomized pilot clinical trial of the safety of pioglitazone in treatment of patients with Alzheimer disease. *Archives of neurology* **68**, 45-50.

[48] Aisen PS, Gauthier S, Ferris SH, Saumier D, Haine D, Garceau D, Duong A, Suhy J, Oh J, Lau WC, Sampalis J (2011) Tramiprosate in mild-to-moderate Alzheimer's disease - A randomized, double-blind, placebo-controlled, multi-centre study (the alphase study). *Archives of Medical Science* **7**, 102-111.

[49] Quinn JF, Raman R, Thomas RG, Yurko-Mauro K, Nelson EB, Van Dyck C, Galvin JE, Emond J, Jack Jr CR, Weiner M, Shinto L, Aisen PS (2010) Docosahexaenoic acid supplementation and cognitive decline in Alzheimer disease: A randomized trial. *JAMA - Journal of the American Medical Association* **304**, 1903-1911.

[50] Maher-Edwards G, Zvartau-Hind M, Hunter AJ, Gold M, Hopton G, Jacobs G, Davy M, Williams P (2010) Double-blind, controlled phase ii study of a 5-ht6 receptor antagonist, sb-742457, in alzheimer's disease. *Current Alzheimer Research* **7**, 374-385.

[51] Gold M, Alderton C, Zvartau-Hind M, Egginton S, Saunders AM, Irizarry M, Craft S, Landreth G, Linnamägi U, Sawchak S (2010) Rosiglitazone monotherapy in mild-to-moderate alzheimer's disease: Results from a randomized, double-blind, placebo-controlled phase III study. *Dementia and Geriatric Cognitive Disorders* **30**, 131-146.

[52] Feldman HH, Doody RS, Kivipelto M, Sparks DL, Waters DD, Jones RW, Schwam E, Schindler R, Hey-Hadavi J, Demicco DA, Breazna A (2010) Randomized controlled trial of atorvastatin in mild to moderate Alzheimer disease: LEADe. *Neurology* **74**, 956-964.

[53] Akhondzadeh S, Sabet MS, Harirchian MH, Togha M, Cheraghmakani H, Razeghi S, Hejazi SS, Yousefi MH, Alimardani R, Jamshidi A, Zare F, Moradi A (2010) Saffron in the treatment of patients with mild to moderate Alzheimer's disease: A 16-week, randomized and placebo-controlled trial. *Journal of Clinical Pharmacy and Therapeutics* **35**, 581-588.

[54] S S, R S, S G, NC F, K B, M R, M S, LS H, R D, CH vD, R M, J B, KM G, E L, I L, D S, R B, M G (2009) A phase 2 multiple ascending dose trial of bapineuzumab in mild to moderate Alzheimer disease. *Neurology* **73**, 2061-2070.

[55] Pasqualetti P, Bonomini C, Dal Forno G, Paulon L, Sinforiani E, Marra C, Zanetti O, Rossini PM (2009) A randomized controlled study on effects of ibuprofen on cognitive progression of Alzheimer's disease. *Aging Clinical and Experimental Research* **21**, 102-110.

[56] Mohs RC, Shiovitz TM, Tariot PN, Porsteinsson AP, Baker KD, Feldman PD (2009) Atomoxetine augmentation of cholinesterase inhibitor therapy in patients with alzheimer disease: 6-month, randomized, double-blind, placebo-controlled, parallel-trial study. *American Journal of Geriatric Psychiatry* **17**, 752-759.

[57] Henderson ST, Vogel JL, Barr LJ, Garvin F, Jones JJ, Costantini LC (2009) Study of the ketogenic agent AC-1202 in mild to moderate Alzheimer's disease: A randomized, double-blind, placebo-controlled, multicenter trial. *Nutrition and Metabolism* **6**.

[58] Hampel H, Ewers M, Bürger K, Annas P, Mörtberg A, Bogstedt A, Frölich L, Schröder J, Schönknecht P, Riepe MW, Kraft I, Gasser T, Leyhe T, Möller HJ, Kurz A, Basun H (2009) Lithium trial in Alzheimer's disease: A randomized, single-blind, placebo-controlled, multicenter 10-week study. *Journal of Clinical Psychiatry* **70**, 922-931.

[59] Green RC, Schneider LS, Amato DA, Beelen AP, Wilcock G, Swabb EA, Zavitz KH (2009) Effect of tarenflurbil on cognitive decline and activities of daily living in patients with mild Alzheimer disease: A randomized controlled trial. *JAMA - Journal of the American Medical Association* **302**, 2557-2564.

[60] Wilcock GK, Black SE, Hendrix SB, Zavitz KH, Swabb EA, Laughlin MA (2008) Efficacy and safety of tarenflurbil in mild to moderate Alzheimer's disease: a randomised phase II trial. *The Lancet Neurology* **7**, 483-493.

[61] Sevigny JJ, Ryan JM, Van Dyck CH, Peng Y, Lines CR, Nessly ML (2008) Growth hormone secretagogue MK-677: No clinical effect on AD progression in a randomized trial. *Neurology* **71**, 1702-1708.

[62] Porsteinsson AP, Grossberg GT, Mintzer J, Olin JT (2008) Memantine treatment in patients with mild to moderate Alzheimer's disease already receiving a cholinesterase inhibitor: A randomized, double-blind, placebo-controlled trial. *Current Alzheimer Research* **5**, 83-89.

[63] Moraes W, Poyares D, Sukys-Claudino L, Guilleminault C, Tufik S (2008) Donepezil improves obstructive sleep apnea in Alzheimer disease: A double-blind, placebo-controlled study. *Chest* **133**, 677-683.

[64] Lannfelt L, Blennow K, Zetterberg H, Batsman S, Ames D, Harrison J, Masters CL, Targum S, Bush AI, Murdoch R, Wilson J, Ritchie CW (2008) Safety, efficacy, and biomarker findings of PBT2 in targeting Aβ as a modifying therapy for Alzheimer's disease: a phase IIa, double-blind, randomised, placebo-controlled trial. *The Lancet Neurology* **7**, 779-786.

[65] Kessler H, Bayer TA, Bach D, Schneider-Axmann T, Supprian T, Herrmann W, Haber M, Multhaup G, Falkai P, Pajonk FG (2008) Intake of copper has no effect on cognition in patients with mild Alzheimer's disease: A pilot phase 2 clinical trial. *Journal of Neural Transmission* **115**, 1181-1187.

[66] Fleisher AS, Raman R, Siemers ER, Becerra L, Clark CM, Dean RA, Farlow MR, Galvin JE, Peskind ER, Quinn JF, Sherzai A, Sowell BB, Aisen PS, Thal LJ (2008) Phase 2 safety trial targeting amyloid β production with a γ-secretase inhibitor in Alzheimer disease. *Archives of Neurology* **65**, 1031-1038.

[67] Doody RS, Gavrilova SI, Sano M, Thomas RG, Aisen PS, Bachurin SO, Seely L, Hung D (2008) Effect of dimebon on cognition, activities of daily living, behaviour, and global function in patients with mild-to-moderate Alzheimer's disease: a randomised, double-blind, placebo-controlled study. *The Lancet* **372**, 207-215.

[68] de Jong D, Jansen R, Hoefnagels W, Jellesma-Eggenkamp M, Verbeek M, Borm G, Kremer B (2008) No effect of one-year treatment with indomethacin on Alzheimer's disease progression: A randomized controlled trial. *PLoS ONE* **3**.

[69] Bakchine S, Loft H (2008) Memantine treatment in patients with mild to moderate Alzheimer's disease: results of a randomised, double-blind, placebo-controlled 6-month study. *J Alzheimers Dis* **13**, 97-107.

[70] Aisen PS, Schneider LS, Sano M, Diaz-Arrastia R, Van Dyck CH, Weiner MF, Bottiglieri T, Jin S, Stokes KT, Thomas RG, Thal LJ (2008) High-dose B vitamin supplementation and cognitive decline in Alzheimer disease: A randomized controlled trial. *JAMA - Journal of the American Medical Association* **300**, 1774-1783.

[71] Winblad B, Cummings J, Andreasen N, Grossberg G, Onofrj M, Sadowsky C, Zechner S, Nagel J, Lane R (2007) A six-month double-blind, randomized, placebo-controlled study of a transdermal patch in Alzheimer's disease - Rivastigmine patch versus capsule. *International Journal of Geriatric Psychiatry* **22**, 456-467.

[72] Soininen H, West C, Robbins J, Niculescu L (2007) Long-term efficacy and safety of celecoxib in Alzheimer's disease. *Dementia and Geriatric Cognitive Disorders* **23**, 8-21.

[73] Feldman HH, Lane R (2007) Rivastigmine: A placebo controlled trial of twice daily and three times daily regimens in patients with Alzheimer's disease. *Journal of Neurology, Neurosurgery and Psychiatry* **78**, 1056-1063.

[74] Chappell AS, Gonzales C, Williams J, Witte MM, Mohs RC, Sperling R (2007) AMPA potentiator treatment of cognitive deficits in Alzheimer disease. *Neurology* **68**, 1008-1012.

[75] Rockwood K, Fay S, Song X, MacKnight C, Gorman M (2006) Attainment of treatment goals by people with Alzheimer's disease receiving galantamine: A randomized controlled trial. *CMAJ* **174**, 1099-1105.

[76] Risner ME, Saunders AM, Altman JFB, Ormandy GC, Craft S, Foley IM, Zvartau-Hind ME, Hosford DA, Roses AD (2006) Efficacy of rosiglitazone in a genetically defined population with mild-to-moderate Alzheimer's disease. *Pharmacogenomics Journal* **6**, 246-254.

[77] Peskind ER, Potkin SG, Pomara N, Ott BR, Graham SM, Olin JT, McDonald S (2006) Memantine treatment in mild to moderate Alzheimer disease: A 24-week randomized, controlled trial. *American Journal of Geriatric Psychiatry* **14**, 704-715.

[78] Moraes Wdos S, Poyares DR, Guilleminault C, Ramos LR, Bertolucci PH, Tufik S (2006) The effect of donepezil on sleep and REM sleep EEG in patients with Alzheimer disease: a double-blind placebo-controlled study. *Sleep* **29**, 199-205.

[79] Sparks DL, Sabbagh MN, Connor DJ, Lopez J, Launer LJ, Browne P, Wasser D, Johnson-Traver S, Lochhead J, Ziolwolski C (2005) Atorvastatin for the treatment of mild to moderate Alzheimer disease: Preliminary results. *Archives of Neurology* **62**, 753-757.

[80] Schneider LS, DeKosky ST, Farlow MR, Tariot PN, Hoerr R, Kieser M (2005) A randomized, double-blind, placebo-controlled trial of two doses of Ginkgo biloba extract in dementia of the Alzheimer's type. *Current Alzheimer Research* **2**, 541-551.

[81] Kennedy J, Deberdt W, Siegal A, Micca J, Degenhardt E, Ahl J, Meyers A, Kaiser C, Baker RW (2005) Olanzapine does not enhance cognition in non-agitated and non-psychotic patients with mild to moderate Alzheimer's dementia. *International Journal of Geriatric Psychiatry* **20**, 1020-1027.

[82] Karaman Y, Erdoǧan F, Köseoǧlu E, Turan T, Ersoy AÖ (2005) A 12-month study of the efficacy of rivastigmine in patients with advanced moderate alzheimer's disease. *Dementia and Geriatric Cognitive Disorders* **19**, 51-56.

[83] Gilman S, Koller M, Black RS, Jenkins L, Griffith SG, Fox NC, Eisner L, Kirby L, Rovira MB, Forette F, Orgogozo JM (2005) Clinical effects of Abeta immunization (AN1792) in patients with AD in an interrupted trial. *Neurology* **64**, 1553-1562.

[84] Brodaty H, Corey-Bloom J, Potocnik FCV, Truyen L, Gold M, Damaraju CRV (2005) Galantamine prolonged-release formulation in the treatment of mild to moderate Alzheimer's disease. *Dementia and Geriatric Cognitive Disorders* **20**, 120-132.

[85] Reines SA, Block GA, Morris JC, Liu G, Nessly ML, Lines CR, Norman BA, Baranak CC (2004) Rofecoxib: no effect on Alzheimer's disease in a 1-year, randomized, blinded, controlled study. *Neurology* **62**, 66-71.

[86] Loeb MB, Molloy DW, Smieja M, Standish T, Goldsmith CH, Mahony J, Smith S, Borrie M, Decoteau E, Davidson W, McDougall A, Gnarpe J, O'Donnell M, Chernesky M (2004) A Randomized, Controlled Trial of Doxycycline and Rifampin for Patients with Alzheimer's Disease. *Journal of the American Geriatrics Society* **52**, 381-387.

[87] Bilikiewicz A, Gaus W (2004) Colostrinin (a naturally occuring, proline-rich, polypeptide mixture) in the treatment of Alzheimer's disease. *Journal of Alzheimer's Disease* **6**, 17-26.

[88] Wolkowitz OM, Kramer JH, Reus VI, Costa MM, Yaffe K, Walton P, Raskind M, Peskind E, Newhouse P, Sack D, De Souza E, Sadowsky C, Roberts E, Schaerf F, Strauss A, Charles L, Wecker N, Segal M (2003) DHEA treatment of Alzheimer's disease: A randomized, double-blind, placebo-controlled study. *Neurology* **60**, 1071-1076.

[89] Tune L, Tiseo PJ, Ieni J, Perdomo C, Pratt RD, Votaw JR, Jewart RD, Hoffman JM (2003) Donepezil HCl (E2020) Maintains Functional Brain Activity in Patients With Alzheimer Disease: Results of a 24-Week, Double-Blind, Placebo-Controlled Study. *American Journal of Geriatric Psychiatry* **11**, 169-177.

[90] Thal LJ, Grundman M, Berg J, Ernstrom K, Margolin R, Pfeiffer E, Weiner MF, Zamrini E, Thomas RG (2003) Idebenone treatment fails to slow cognitive decline in Alzheimer's disease. *Neurology* **61**, 1498-1502.

[91] Moreno Moreno MDJ (2003) Cognitive improvement in mild to moderate Alzheimer's dementia after treatment with the acetylcholine precursor choline alfoscerate: A multicenter, double-blind, randomized, placebo-controlled trial. *Clinical Therapeutics* **25**, 178-193.

[92] Krishnan KRR, Charles HC, Doraiswamy PM, Mintzer J, Weisler R, Yu X, Perdomo C, Ieni JR, Rogers S (2003) Randomized, placebo-controlled trial of the effects of donepezil on neuronal markers and hippocampal volumes in Alzheimer's disease. *American Journal of Psychiatry* **160**, 2003-2011.

[93] Akhondzadeh S, Noroozian M, Mohammadi M, Ohadinia S, Jamshidi AH, Khani M (2003) Melissa officinalis extract in the treatment of patients with mild to moderate Alzheimer's disease: A double blind, randomised, placebo controlled trial. *Journal of Neurology Neurosurgery and Psychiatry* **74**, 863-866.

[94] Akhondzadeh S, Noroozian M, Mohammadi M, Ohadinia S, Jamshidi AH, Khani M (2003) Salvia officinalis extract in the treatment of patients with mild to moderate Alzheimer's disease: A double blind, randomized and placebo-controlled trial. *Journal of Clinical Pharmacy and Therapeutics* **28**, 53-59.

[95] Aisen PS, Schafer KA, Grundman M, Pfeiffer E, Sano M, Davis KL, Farlow MR, Jin S, Thomas RG, Thal LJ (2003) Effects of Rofecoxib or Naproxen vs Placebo on Alzheimer Disease Progression: A Randomized Controlled Trial. *Journal of the American Medical Association* **289**, 2819-2826.

[96] Ruether E, Alvarez XA, Rainer M, Moessler H (2002) Sustained improvement of cognition and global function in patients with moderately severe Alzheimer's disease: a double-blind, placebo-controlled study with the neurotrophic agent Cerebrolysin. *J Neural Transm Suppl*, 265-275.

[97] Panisset M, Gauthier S, Moessler H, Windisch M (2002) Cerebrolysin in Alzheimer's disease: A randomized, double-blind, placebo-controlled trial with a neurotrophic agent. *Journal of Neural Transmission* **109**, 1089-1104.

[98] M S, F S, D L, K vB, K B, J D, H W, T H, JB S (2002) Treatment with simvastatin in normocholesterolemic patients with Alzheimer's disease: A 26-week randomized, placebo-controlled, double-blind trial. *Annals of neurology* **52**, 346-350.

[99] Winblad B, Bonura ML, Rossini BM, Battaglia A (2001) Nicergoline in the treatment of mild-to-moderate Alzheimer's disease: A European multicentre trial. *Clinical Drug Investigation* **21**, 621-632.

[100] Wilkinson D, Murray J (2001) Galantamine: A randomized, double-blind, dose comparison in patients with Alzheimer's disease. *International Journal of Geriatric Psychiatry* **16**, 852-857.

[101] Ruether E, Husmann R, Kinzler E, Diabl E, Klingler D, Spatt J, Ritter R, Schmidt R, Taneri Z, Winterer W, Koper D, Kasper S, Rainer M, Moessler H (2001) A 28-week, double-blind, placebo-controlled study with Cerebrolysin in patients with mild to moderate Alzheimer's disease. *International Clinical Psychopharmacology* **16**, 253-263.

[102] Rockwood K, Mintzer J, Truyen L, Wessel T, Wilkinson D (2001) Effects of a flexible galantamine dose in Alzheimer's disease: A randomised, controlled trial. *Journal of Neurology Neurosurgery and Psychiatry* **71**, 589-595.

[103] Wilcock GK, Lilienfeld S, Gaens E (2000) Efficacy and safety of galantamine in patients with mild to moderate Alzheimer\"s disease: multicentre randomised controlled trial. *Bmj* **321**, 1445-1449.

[104] Van Dyck CH, Newhouse P, Falk WE, Mattes JA (2000) Extended-release physostigmine in Alzheimer disease: A multicenter, double-blind, 12-week study with dose enrichment. *Archives of General Psychiatry* **57**, 157-164.

[105] Thal LJ, Forrest M, Loft H, Mengel H (2000) Lu 25-109, a muscarinic agonist, fails to improve cognition in Alzheimer's disease. *Neurology* **54**, 421-426.

[106] Thal LJ, Calvani M, Amato A, Carta A (2000) A 1-year controlled trial of acetyl-L-carnitine in early-onset AD. *Neurology* **55**, 805-810.

[107] Tariot PN, Solomon PR, Morris JC, Kershaw P, Lilienfeld S, Ding C (2000) A 5-month, randomized, placebo-controlled trial of galantamine in AD. *Neurology* **54**, 2269-2276.

[108] Raskind MA, Peskind ER, Wessel T, Yuan W (2000) Galantamine in AD: A 6-month randomized, placebo-controlled trial with a 6-month extension. *Neurology* **54**, 2261-2268.

[109] Imbimbo BP, Troetel WM, Martelli P, Lucchelli F (2000) A 6-month, double-blind, placebo-controlled trial of eptastigmine in Alzheimer's disease. *Dementia and Geriatric Cognitive Disorders* **11**, 17-24.

[110] Homma A, Takeda M, Imai Y, Udaka F, Hasegawa K, Kameyama M, Nishimura T (2000) Clinical efficacy and safety of donepezil on cognitive and global function in patients with Alzheimer's disease: A 24-week, multicenter, double-blind, placebo-controlled study in Japan. *Dementia and Geriatric Cognitive Disorders* **11**, 299-313.

[111] Bae CY, Cho CY, Cho K, Hoon Oh B, Choi KG, Lee HS, Jung SP, Kim DH, Lee S, Choi GD, Cho H, Lee H (2000) A double-blind, placebo-controlled, multicenter study of Cerebrolysin for Alzheimer's disease. *Journal of the American Geriatrics Society* **48**, 1566-1571.

[112] Thal LJ, Ferguson JM, Mintzer J, Raskin A, Targum SD (1999) A 24-week randomized trial of controlled-release physostigmine in patients with Alzheimer's disease. *Neurology* **52**, 1146-1152.

[113] S S, A M, A U, F V, N C (1999) A double-blind, placebo-controlled trial of diclofenac/misoprostol in Alzheimer's disease. *Neurology* **53**, 197-201.

[114] Rösler M, Anand R, Cicin-Sain A, Gauthier S, Gharabawi M (1999) Efficacy and safety of rivastigmine in patients with Alzheimer's disease: International randomised controlled trial. *Bmj* **318**, 633-638.

[115] Jann MW, Cyrus PA, Eisner LS, Margolin DI, Griffin T, Gulanski B (1999) Efficacy and safety of a loading-dose regimen versus a no-loading-dose regimen of metrifonate in the symptomatic treatment of Alzheimer's disease: A randomized, double-masked, placebo-controlled trial. *Clinical Therapeutics* **21**, 88-102.

[116] Forette F, Anand R, Gharabawi G (1999) A phase II study in patients with Alzheimer's disease to assess the preliminary efficacy and maximum tolerated dose of rivastigmine (Exelon®). *European Journal of Neurology* **6**, 423-429.

[117] Dubois B, McKeith I, Orgogozo JM, Collins O, Meulien D (1999) A multicentre, randomized, double-blind, placebo-controlled study to evaluate the efficacy, tolerability and safety of two doses of metrifonate in patients with mild-to-moderate Alzheimer's disease: The MALT study. *International Journal of Geriatric Psychiatry* **14**, 973-982.

[118] Burns A, Rossor M, Hecker J, Gauthier S, Petit H, Möller HJ, Rogers SL, Friedhoff LT (1999) The effects of donepezil in Alzheimer's disease - Results from a multinational trial. *Dementia and Geriatric Cognitive Disorders* **10**, 237-244.

[119] Álvarez XA, Mouzo R, Pichel V, Pérez P, Laredo M, Fernández-Novoa L, Corzo L, Zas R, Alcaraz M, Secades JJ, Lozano R, Cacabelos R (1999) Double-blind placebo-controlled study with citicoline in APOE genotyped Alzheimer's disease patients. Effects on cognitive performance, brain bioelectrical activity and cerebral perfusion. *Methods and Findings in Experimental and Clinical Pharmacology* **21**, 633-644.

[120] Rogers SL, Farlow MR, Doody RS, Mohs R, Friedhoff LT (1998) A 24-week, double-blind, placebo-controlled trial of donepezil in patients with Alzheimer's disease. *Neurology* **50**, 136-145.

[121] Rogers SL, Doody RS, Mohs RC, Friedhoff LT (1998) Donepezil improves cognition and global function in Alzheimer disease: A 15-week, double-blind, placebo-controlled study. *Archives of Internal Medicine* **158**, 1021-1031.

[122] Morris JC, Cyrus PA, Orazem J, Mas J, Bieber F, Ruzicka BB, Gulanski B (1998) Metrifonate benefits cognitive, behavioral, and global function in patients with Alzheimer's disease. *Neurology* **50**, 1222-1230.

[123] Imbimbo BP, Lucca U, Lucchelli F, Alberoni M, Thal LJ (1998) A 25-week placebo-controlled study of eptastigmine in patients with Alzheimer disease. *Alzheimer Disease and Associated Disorders* **12**, 313-322.

[124] Cummings JL, Cyrus PA, Bieber F, Mas J, Orazem J, Gulanski B (1998) Metrifonate treatment of the cognitive deficits of Alzheimer's disease. *Neurology* **50**, 1214-1221.

[125] Weyer G, Babej-Dölle RM, Hadler D, Hofmann S, Herrmann WM (1997) A controlled study of 2 doses of idebenone in the treatment of Alzheimer's disease. *Neuropsychobiology* **36**, 73-82.

[126] Rockwood K, Beattie BL, Eastwood MR, Feldman H, Mohr E, Pryse-Phillips W, Gauthier S (1997) A randomized, controlled trial of linopirdine in the treatment of Alzheimer's disease. *Canadian Journal of Neurological Sciences* **24**, 140-145.

[127] Bodick NC, Offen WW, Levey AI, Cutler NR, Gauthier SG, Satlin A, Shannon HE, Tollefson GD, Rasmussen K, Bymaster FP, Hurley DJ, Potter WZ, Paul SM (1997) Effects of xanomeline, a selective muscarinic receptor agonist, on cognitive function and behavioral symptoms in Alzheimer disease. *Archives of Neurology* **54**, 465-473.

[128] Weyer G, Erzigkeit H, Hadler D, Kubicki S (1996) Efficacy and safety of idebenone in the long-term treatment of Alzheimer's disease: A double-blind, placebo controlled multicentre study. *Human Psychopharmacology* **11**, 53-65.

[129] Shrotriya RC, Cutler NR, Sramek JJ, Veroff AE, Hironaka DY (1996) Efficacy and safety of BMY 21,502 in Alzheimer disease. *Annals of Pharmacotherapy* **30**, 1376-1380.

[130] Rogers S, Friedhoff LT, Apter JT, Richter RW, Hartford JT, Walshe TM, Baumel B, Linden RD, Cleveland Kinney F, Doody RS, Borison RL, Ahem GL, Rogers SL (1996) The efficacy and safety of donepezil in patients with Alzheimer's disease: Results of a US multicentre, randomized, double-blind, placebo-controlled trial. *Dementia* **7**, 293-303.

[131] Huff FJ, Antuono PG, Delagandara JE, McDonald MA, Cutler NR, Cohen SR, Green RC, Zemlan FP, Crismon ML, Alter M, Shipley JE, Reichman WE (1996) A treatment and withdrawal trial of besipirdine in Alzheimer disease. *Alzheimer Disease and Associated Disorders* **10**, 93-102.

[132] Becker RE, Colliver JA, Markwell SJ, Moriearty PL, Unni LK, Vicari S (1996) Double-blind, placebo-controlled study of metrifonate, an acetylcholinesterase inhibitor, for Alzheimer disease. *Alzheimer Disease and Associated Disorders* **10**, 124-131.

[133] Knapp MJ, Knopman DS, Solomon PR, Pendlebury WW, Davis CS, Gracon SI (1994) A 30-week randomized controlled trial of high-dose tacrine in patients with Alzheimer's disease. *Journal of the American Medical Association* **271**, 985-991.

[134] Farlow M, Gracon SI, Hershey LA, Lewis KW, Sadowsky CH, Dolan-Ureno J (1992) A controlled trial of tacrine in Alzheimer's disease. The Tacrine Study Group. *Jama* **268**, 2523-2529.

**Table S1** Baseline information of the included AD trials

| Source | Registration number | Disease type | Trial location | Number of sites | Duration, week | Add-on design | Randomized  (Completed) | Analyzed  size | Analyzed dataset | JADAD  score | Age,  year | Male,  % | Background  therapy, % | APOEε4  Carrier, % | ADAS_cog_11  point | MMSE,  point |
| --- | --- | --- | --- | --- | --- | --- | --- | --- | --- | --- | --- | --- | --- | --- | --- | --- |
| Vila-Castelar 2019 | NCT03073876 | probable AD | USA | 1 | 6 | N | 13 (11) | 11 | PP | 6 | 81.7 | 36.4 | 0 | NA | 13.1 | 25.4 |
| Bernard 2019 | NCT02626572 | mild to moderate AD | International | 75 | 24 | N | 129 (119) | 129 | FAS | 4 | 71.4 | 30.2 | 0 | 46.9 | 23.26 | 19.7 |
| Zhu 2018 | NCT00678431 | probable or possible AD | USA | 1 | 52 | N | 15 (13) | 13 | ITT | 5 | 79.3 | 61.5 | NA | NA | 29.2 | 19.4 |
| Wang 2018 | NA | AD | China | 1 | 6 | N | 21 (21) | 21 | ITT | 7 | 74.6 | 23.8 | NA | NA | 29.7 | NA |
| Voss 2018 | NCT01852110 | AD | USA and Canada | 59 | 24 | Y | 120 (74) | 99 | FAS | 5 | 71.7 | 43.3 | 100 | 54.2 | 23.6 | 18.3 |
| Rafii 2018 | NCT00876863 | mild to moderate AD | USA | 10 | 104 | Y | 23 (19) | 23 | ITT | 7 | 68 | 54 | 100 | 69 | 20.3 | 22.1 |
| Fullerton 2018 | NCT01712074 | mild to moderate AD | USA and Europe | 37 | 12 | Y | 94 (86) | 87 | FAS | 5 | 75.9 | 41.5 | 100 | 51.2 | 24.1 | 19.6 |
| Egan 2018 | NCT01739348 | mild to moderate AD | International | 238 | 78 | N | 653 (471) | 639 | FAS | 6 | 72.4 | 45.8 | 89.3 | 63.6 | 21.7 | 20.3 |
| Atri 2018 | NCT01955161 | mild to moderate AD | International | 119 | 24 | Y | 310 (285) | 304 | FAS | 6 | 73.7 | 36.2 | 100 | 61.3 | 25.8 | 17.4 |
|  | NCT02006641 | mild to moderate AD | International | 158 | 24 | Y | 284 (260) | 278 | FAS | 6 | 73.5 | 35.8 | 100 | 57.4 | 25.7 | 17.6 |
|  | NCT02006654 | mild to moderate AD | International | 126 | 24 | Y | 369 (328) | 356 | FAS | 6 | 74.2 | 36.5 | 100 | 58.5 | 25.9 | 17.5 |
| Relkin 2017 | NCT00818662 | mild to moderate AD | USA and Canada | 41 | 78 | Y | 123 (98) | 126 | ITT | 4 | 70.2 | 46.3 | 100 | 69.1 | 23.1 | 21.1 |
| Nave 2017 | NCT01677754 | moderate AD | International | >100 | 52 | Y | 181 (147) | 181 | ITT | 5 | 73.8 | 42.5 | 100 | 57.5 | 26.6 | 17.5 |
| Gauthier 2016 | NCT01689246/EudraCT 21012-002847-28 | mild to moderate AD | International | 115 | 65 | N | 357 (271) | 348 | mITT | 7 | 70.7 | 38 | 87 | 48 | 27.2 | 18.6 |
| Gault 2016 | NCT01527916 | mild to moderate AD | International | 33 | 24 | N | 104 (89) | 98 | ITT | 6 | 73.2 | 37.5 | 0 | 40.2 | 26.1 | 19.1 |
| Florian 2016 | NCT01549834 | mild to moderate AD | International | 43 | 24 | Y | 146 (129) | 144 | ITT | 6 | 75.1 | 50.7 | 100 | 62.2 | 24.4 | 18.7 |
| Choe 2016 | NA | mild to moderate AD | Korea | 4 | 52 | Y | 37 (34) | 37 | ITT | 5 | 75.4 | 27 | 100 | 54.1 | 23.85 | 15.62 |
| Tajadini 2015 | IRCT2014010216028N1 | mild to moderate AD | Iran | 1 | 12 | N | 25 (20) | 25 | NA | 4 | 67.65 | 50 | NA | NA | 17.1 | NA |
| Maher-Edwards 2015 | NCT00708552 | mild to moderate AD | International | 68 | 24 | N | 145 (118) | 135 | ITT | 5 | 73.3 | 36 | 0 | NA | 29.1 | 18.2 |
|  | NCT00710684 | mild to moderate AD | International | 97 | 48 | Y | 226 (198) | 223 | ITT | 6 | 73.1 | 42 | 100 | NA | 27.1 | 18.4 |
| Lenz 2015 | NCT00555204 | mild to moderate AD | USA | 41 | 12 | Y | 102 (61) | 101 | NA | 5 | 75 | 40.6 | 100 | NA | 20.2 | 20.4 |
| Henderson 2015 | NCT00368459 | mild to moderate AD | USA | 3 | 52 | Y | 21 (20) | 21 | ITT | 7 | 74.1 | 0 | 100 | NA | 25.8 | 19.4 |
| Gauthier 2015 | NCT00842816 | AD | USA and Canada | 31 | 12 | Y | 51 (43) | 43 | PP | 5 | 78.3 | 58.8 | 100 | NA | 24.3 | 17.3 |
| Gault 2015 | NCT00948909 | mild to moderate AD | International | 27 | 12 | N | 68 (65) | 67 | ITT | 7 | 73.6 | 38.2 | 0 | 55.9 | 24.7 | 19.7 |
| Bowen 2015 | NA | mild to moderate AD | USA | 5 | 48 | N | 36 (25) | 36 | ITT | 3 | 77.5 | 0 | 72.2 | 91.7 | 21.9 | 17.9 |
| Wilkinson 2014 | NCT01019421 | moderate AD | International | 48 | 24 | Y | 133 (118) | 132 | FAS | 7 | 75 | 33 | 100 | NA | 28 | 17 |
| Shinto 2014 | NCT00090402 | AD | USA | NA | 52 | N | 13 (11) | 13 | NA | 5 | 75.2 | 46 | 77 | NA | 32.2 | 22.2 |
| Schwam 2014 | NA | mild to moderate AD | International | 36 | 12 | N | 100 (92) | 100 | FAS | 4 | 73.5 | 36 | 0 | NA | 21.9 | 20.3 |
| Salloway 2014 | NCT00575055 | mild to moderate AD | USA | 170 | 78 | N | 448 (338) | 432 | mITT | 6 | 72.3 | 44 | 92.6 | 100 | 23.9 | 20.7 |
|  | NCT00574132 | mild to moderate AD | International | 218 | 78 | N | 524 (373) | 493 | mITT | 6 | 71.9 | 49.7 | 89.7 | 0 | 22.2 | 21.2 |
| Marek 2014 | NCT01137526 | mild to moderate AD | International | 30 | 12 | N | 66 (44) | 64 | ITT | 6 | 71.7 | 39.4 | 0 | NA | 25.8 | 19.4 |
| Haig 2014 | NCT01018875 | mild to moderate AD | Russia and Ukraine | 21 | 12 | N | 63 (38) | 56 | ITT | 7 | 70.3 | 38.1 | 0 | 52.8 | 31.9 | 18.2 |
| Grove 2014 | NCT01009255 | mild to moderate AD | International | 34 | 16 | N | 99 (92) | 92 | ITT | 7 | 72 | 30 | 0 | NA | 25.5 | 19.9 |
| Grimaldi 2014 | NCT01075763 | mild to moderate AD | Italy | 5 | 28 | N | 19 (16) | 19 | NA | 4 | 64.57 | 42.1 | NA | NA | 19.81 | 22.93 |
| Galasko 2014 | NCT00566397 | mild to moderate AD | USA | 40 | 52+26 | Y | 132 (NA) | 132 | multiple imputation | 6 | 72.2 | 43 | 100 | 74 | 24.1 | 20.5 |
| Dysken 2014 | NCT00235716 | mild to moderate AD | USA | 14 | 208 | Y | 152 (90) | 152 | NA | 7 | 79.4 | 98 | 100 | 46.6 | 19.1 | 20.8 |
| Doody 2014 | NCT00905372 | mild to moderate AD | International | NA | 78 | N | 506 (370) | 506 | ITT | 6 | 74.4 | 43.3 | 88.3 | 61.3 | 22 | 21 |
|  | NCT00904683 | mild to moderate AD | International | NA | 78 | N | 519 (400) | 517 | ITT | 6 | 72.4 | 44.9 | 91.7 | 59.5 | 23 | 21 |
| Shah 2013 | NTR1683 | mild to moderate AD | USA | 48 | 24 | Y | 262 (223) | 262 | ITT | 6 | 76.9 | 48 | 100 | 58 | 23.39 | 19.4 |
| Molloy 2013 | NA | mild to moderate AD | Canada | 4 | 52 | N | 102 (99) | 102 | NA | 6 | 78.6 | 51 | 94.1 | NA | 22 | 22 |
| Doody 2013 | NCT00594568 | mild to moderate AD | International | NA | 76 | N | 501 (189) | 486 | ITT | 7 | 73.3 | 45 | 87 | 58 | 22.8 | 20.9 |
| Egan 2012 | NCT00420420 | mild to moderate AD | USA | 15 | 4 | N | 71 (67) | 70 | FAS | 7 | 73.78 | 49.3 | 87.3 | 50.7 | 18.4 | 22.2 |
| Coric 2012 | NCT00810147 | mild to moderate AD | International | NA | 24 | N | 42 (34) | 42 | NA | 5 | 73.7 | 40.5 | 85.7 | 57.1 | 20.4 | 21.5 |
| Andersen 2012 | NCT00443014 | mild to moderate AD | Norway | 9 | 52 | N | 40 (NA) | 40 | NA | 3 | 79.81 | 46 | 0 | NA | 17.2 | 23.3 |
| Vellas 2011 | NCT00880412 | mild to moderate AD | France | 23 | 12 | Y | 53 (51) | 52 | ITT | 5 | 75.8 | 47.2 | 100 | 51.1 | NA | 19.8 |
| Sano 2011 | NCT00053599 | mild to moderate AD | USA | 45 | 78 | N | 202 (162) | 202 | ITT | 7 | 75.1 | 40.1 | 94.06 | 55.3 | 23.9 | 20.7 |
| Rafii 2011 | NCT00083590 | mild to moderate AD | USA | NA | 16 | N | 73 (64) | 69 | ITT | 4 | 78.1 | 35.62 | 43 | 65.15 | 27.1 | 19.12 |
| Nakamura 2011 | NCT00423085 | AD | Japan | NA | 24 | N | 288 (242) | 268 | ITT | 7 | 74.5 | 31.8 | 0 | NA | 25.1 | 16.6 |
| Maher-Edwards 2011 | NCT00348192 | mild to moderate AD | International | 24 | 24 | N | 63 (46) | 56 | ITT | 5 | 71.6 | 30 | 0 | NA | 27.7 | 18.3 |
| Harrington 2011 | NCT00348309 | mild to moderate AD | International | 228 | 48 | Y | 461 (348) | 461 | ITT | 4 | 74 | 39 | 100 | 56 | 25.3 | 19.6 |
|  | NCT00348140 | mild to moderate AD | International | 184 | 48 | Y | 479 (340) | 479 | ITT | 4 | 72.8 | 44 | 100 | 59 | 24.3 | 19.8 |
| Frölich 2011 | NCT00501111 | mild to moderate AD | Europe | 84 | 12 | N | 164 (145) | 157 | ITT | 4 | 73.5 | 44.8 | 0 | NA | 24 | 21 |
| Geldmacher 2011 | NCT00982202 | AD | USA | 2 | 78 | N | 15 (0) | 15 | NA | 5 | 67 | 40 | NA | NA | 20.2 | 21.3 |
| Aisen 2011 | NA | mild to moderate AD | USA and Canada | 67 | 78 | Y | 353 (253) | 331 | ITT | 7 | 74.2 | 46.6 | 100 | 64.7 | 22.2 | 21 |
| Quinn 2010 | NCT00440050 | mild to moderate AD | USA | 51 | 78 | N | 164 (124) | 164 | ITT | 7 | 76 | 40.2 | 83.5 | 57.9 | 23.96 | 20.3 |
| Maher-Edwards 2010 | NCT00224497 | mild to moderate AD | International | 58 | 24 | N | 124 (102) | 119 | ITT | 6 | 70.5 | 38 | NA | NA | 24.1 | 20.5 |
| Gold 2010 | NCT00428090 | mild to moderate AD | International | 134 | 24 | N | 166 (131) | 131 |  | 5 | 72.5 | 40 | NA | 48 | 25 | 19.6 |
| Feldman 2010 | NCT00053599 | mild to moderate AD | International | 87 | 72 | Y | 326 (245) | 317 | ITT | 6 | 73.2 | 49 | 100 | NA | 22.5 | 21.9 |
| Akhondzadeh 2010 | NA | mild to moderate AD | Iran | NA | 16 | N | 23 (20) | 23 | ITT | 7 | 73.13 | 52.1 | NA | NA | 25 | NA |
| Salloway 2009 | NCT00112073 | mild to moderate AD | USA | 30 | 78 | N | 110 (78) | 107 | mITT | 5 | 67.9 | 40.2 | 96.3 | 69.8 | NA | 20.7 |
| Pasqualetti 2009 | NA | mild to moderate AD | Italy | 7 | 52 | N | 66 (46) | 66 | ITT | 7 | 74 | 35 | NA | NA | 25.6 | 20.3 |
| Mohs 2009 | NA | mild to moderate AD | USA | 8 | 26 | Y | 45 (35) | 44 | ITT | 5 | 77.4 | 45.7 | 100 | NA | 21.3 | 20.3 |
| Henderson 2009 | NCT00142805 | mild to moderate AD | USA | 23 | 15 | N | 66 (0) | 63 | ITT | 5 | 76.8 | 47 | 83.3 | 54.4 | 23.35 | 19.48 |
| Hampel 2009 | ISRCTN72046462 | mild AD | Germany | 6 | 10 | N | 38 (37) | 38 | NA | 4 | 68.9 | 50 | 24 | NA | 15.4 | 23.6 |
| Green 2009 | NCT00105547 | mild AD | USA | 133 | 78 | N | 822 (540) | 746 | ITT | 7 | 74.7 | 47.5 | 82 | 57.9 | 17.8 | 23.3 |
| Wilcock 2008 | 20365/0001/A 69316 | mild to moderate AD | Canada and UK | 31 | 52 | N | 71 (56) | 46 | ITT | 7 | 75.6 | 59 | 98 | NA | 27.5 | 22.9 |
| Sevigny 2008 | NCT00074529 | AD | USA | 45 | 52 | N | 281 (209) | 280 | ITT | 7 | 76.1 | 40.2 | 71.2 | 41.3 | 22.2 | 20.6 |
| Porsteinsson 2008 | NA | mild to moderate AD | USA | 38 | 24 | Y | 216 (191) | 212 | ITT | 7 | 76 | 49.5 | 100 | NA | 26.8 | 17 |
| Moraes 2008 | NCT00480870 | mild to moderate AD | Brazil | 1 | 12 | N | 12 (NA) | 12 | ITT | 6 | 72.6 | 41.7 | 0 | NA | 29.3 | 17.2 |
| Lannfelt 2008 | NCT00471211 | early AD | Sweden and Australia | 15 | 12 | Y | 29 (28) | 29 | ITT | 7 | 71.6 | 48 | 100 | 72 | 18.9 | 22.2 |
| Kessler 2008 | NCT00608946 | mild AD | Germany | NA | 52 | Y | 33 (28) | 28 | NA | 5 | 69.4 | 35.7 | 100 | NA | 17.6 | NA |
| Fleisher 2008 | NCT00244322 | mild to moderate AD | USA | 6 | 14 | N | 15 (12) | 12 | PP | 5 | 68.7 | 67 | 87 | 67 | 27 | 18.9 |
| Doody 2008 | NCT00377715 | mild to moderate AD | Russia | 11 | 52 | N | 94 (NA) | 94 | ITT | 7 | 68.4 | 38 | 0 | NA | NA | 18.3 |
| de 2008 | NCT00432081 | AD | Netherland | 2 | 52 | N | 25 (19) | 25 | ITT | 7 | 72.2 | 24 | 8 | 44 | 19.7 | 20.2 |
| Bakchine 2008 | NA | mild to moderate AD | International | 65 | 24 | N | 152 (138) | 135 | PP | 7 | 73.3 | 40 | 0 | NA | 24.9 | 18.9 |
| Aisen 2008 | NCT00056225 | mild to moderate AD | USA | 40 | 78 | N | 169 (140) | 166 | ITT | 7 | 77.3 | 46.1 | 91.2 | 70 | 22.63 | 20.91 |
| Winblad 2007 | NA | AD | International | 100 | 24 | N | 302 (266) | 281 | ITT | 5 | 73.9 | 33.4 | NA | NA | 28.6 | 16.4 |
| Soininen 2007 | IQ5-97-02-001 | AD | International | 30 | 52 | N | 140 (108) | 140 | ITT | 5 | 73.3 | 41.4 | NA | NA | 24.6 | 19.4 |
| Feldman 2007 | NA | mild to moderate AD | International | 37 | 26 | N | 222 (189) | 220 | ITT | 5 | 71.7 | 40 | NA | NA | 28.5 | 18.7 |
| Chappell 2007 | NA | mild to moderate AD | USA | 18 | 11 | N | 91 (85) | 91 | ITT | 7 | 74.5 | 53 | 0 | NA | 19 | 20.5 |
| Rockwood 2006 | NA | mild to moderate AD | Canada | 14 | 16 | N | 66 (56) | 65 | ITT | 6 | 78 | 38 | NA | NA | 27.9 | 19.9 |
| Risner 2006 | NA | mild to moderate AD | Europe and New Zealand | 67 | 24 | N | 122 (106) | 122 | ITT | 6 | 71.8 | 37 | 0 | NA | NA | 20.8 |
| Peskind 2006 | NA | mild to moderate AD | USA | 42 | 24 | N | 202 (167) | 198 | ITT | 7 | 77 | 42.6 | NA | NA | 27.3 | 17.2 |
| Moraes 2006 | NA | mild to moderate AD | Brazil | 2 | 26 | N | 18 (18) | 18 | NA | 4 | 74.5 | 38.9 | NA | NA | 39 | NA |
| Sparks 2005 | NA | mild to moderate AD | USA | NA | 52 | N | 31 (21) | 31 | ITT | 6 | 78.9 | 64.5 | 9.7 | 60 | 19.9 | 20.52 |
| Schneider 2005 | NA | AD | USA | 44 | 26 | N | 174 (135) | 174 | ITT | 6 | 77.5 | 48 | 0 | NA | 25 | 18.2 |
| Kennedy 2005 | NA | mild to moderate AD | USA | NA | 26 | N | 90 (66) | 90 | ITT | 4 | 78 | 42.2 | 0 | NA | 21.03 | 21.47 |
| Karaman 2005 | NA | advanced moderate AD | Turkey | 1 | 52 | N | 20 (NA) | 20 | NA | 4 | 73.4 | 45 | NA | NA | 39.3 | 13.2 |
| Gilman 2005 | NA | AD | USA and Europe | 28 | 52 | N | 73 (53) | 65 | NA | 5 | 71 | 40.3 | 86.1 | 58.6 | 23.9 | 20.2 |
| Brodaty 2005 | NA | mild to moderate AD | International | 93 | 26 | N | 324 (266) | 305 | ITT | 6 | 76.3 | 36 | 0 | NA | 26.1 | 18.08 |
| Reines 2004 | NA | mild to moderate AD | USA | 31 | 52 | N | 346 (0) | 327 |  | 7 | 75 | 48 | 58 | NA | 20 | 21 |
| Loeb 2004 | NA | AD | Canada | 5 | 52 | N | 50 (39) | 47 | ITT | 6 | 75 | 40 | 84 | 53 | 28.6 | 19.1 |
| Bilikiewicz 2004 | NA | mild to moderate AD | Poland | 6 | 15 | N | 52 (NA) | 52 | NA | 6 | 72.1 | 33.3 | NA | NA | 27.5 | NA |
| Wolkowitz 2003 | NA | AD | USA | 9 | 26 | N | 30 (14) | 22 | NA | 6 | 77.2 | 53 | 0 | NA | 22.9 | 21.9 |
| Tune 2003 | NA | mild to moderate AD | USA | 1 | 24 | N | 14 (12) | 14 | NA | 4 | 72.2 | 28.6 | 0 | NA | 21.81 | 21.4 |
| Thal 2003 | NA | AD | USA | 39 | 52 | N | 129 (96) | 129 | ITT | 6 | 74.9 | 49 | 3.2 | 60 | 21.7 | 20.5 |
| Moreno 2003 | NA | mild to moderate AD | Mexico | 5 | 26 | N | 129 (114) | 129 | ITT | 5 | 71.7 | 27.1 | 0 | NA | 36.74 | 17.62 |
| Krishnan 2003 | NA | mild to moderate AD | USA | 3 | 24 | N | 34 (28) | 32 | ITT | 6 | 72.4 | 30 | 0 | NA | 26.44 | 19 |
| Akhondzadeh 2003 | NA | mild to moderate AD | Iran | 3 | 16 | N | 20 (15) | 15 | PP | 6 | 72.75 | 60 | 0 | NA | 25.6 | NA |
| Akhondzadeh 2003 | NA | mild to moderate AD | Iran | 3 | 16 | N | 21 (15) | 15 | PP | 4 | 73.7 | 57.1 | 0 | NA | 27.9 | NA |
| Aisen 2003 | NA | mild to moderate AD | USA | 40 | 52 | N | 111 (88) | 111 | ITT | 7 | 73.8 | 44.1 | 67 | 67.6 | 24.2 | 20.8 |
| Ruether 2002 | NA | more advanced AD | Germany and Austrilia | 9 | 28 | N | 49 (NA) | 49 | ITT | 5 | 73.5 | 42.8 | NA | NA | 35.8 | 15.5 |
| Panisset 2002 | NA | AD | Canada | 14 | 26 | N | 95 (83) | 89 | ITT | 6 | 75.19 | 44 | NA | NA | 23.63 | 20.93 |
| Simons 2002 | NA | AD | Germany | NA | 26 | N | 20 (17) | 20 | NA | 5 | 68.5 | 53 | NA | NA | 33.2 | 17.1 |
| Winblad 2001 | NA | mild to moderate AD | Europe | 33 | 26 | N | 169 (137) | 169 | ITT | 5 | 73.7 | 36.1 | 0 | NA | 28.3 | 18.5 |
| Wilkinson 2001 | NA | mild to moderate AD | UK | 8 | 12 | N | 87 (73) | 82 | ITT | 7 | 74.2 | 41 | NA | NA | 26.9 | 18.7 |
| Ruether 2001 | NA | mild to moderate AD | Germany and Austrilia | 9 | 28 | N | 73 (66) | 70 | ITT | 7 | 73.5 | 48.6 | NA | NA | 30.2 | 17.5 |
| Rockwood 2001 | NA | AD | International | 43 | 12 | N | 125 (113) | 108 | PP | 7 | 74.6 | 46.4 | 0 | 64.8 | 24.7 | 19.6 |
| Wilcock 2000 | NA | mild to moderate AD | Canada and Europe | 86 | 24 | N | 215 (186) | 171 | PP | 7 | 72.7 | 38.6 | 0 | 63 | 24.7 | 19.3 |
| Van 2000 | NA | mild to moderate AD | USA | 36 | 12 | N | 93 (82) | 90 | ITT | 6 | 71.4 | 67 | NA | NA | 29 | 18.4 |
| Thal 2000 | NA | mild to moderate AD | USA | 29 | 24 | N | 126 (93) | 126 | ITT | 6 | 76 | 44.4 | NA | 53 | 22.7 | 20.1 |
| Thal 2000 | NA | AD | USA | NA | 52 | N | 117 (88) | 102 | ITT | 5 | 58 | 53 | 0 | NA | 22.9 | 20.6 |
| Tariot 2000 | NA | mild to moderate AD | USA | NA | 20 | N | 286 (240) | 225 | PP | 6 | 77.1 | 37.8 | 0 | 64.7 | 29.4 | 17.7 |
| Raskind 2000 | NA | mild to moderate AD | USA | 33 | 26 | N | 213 (172) | 157 | PP | 6 | 75.3 | 38.5 | 0 | 58.2 |  | 19.2 |
| Imbimbo 2000 | NA | mild to moderate AD | Italy and USA | 26 | 25 | N | 119 (98) | 114 | ITT | 3 | 72.3 | 34 | NA | NA | 30.9 | 17.8 |
| Homma 2000 | NA | AD | Japan | 54 | 24 | N | 129 (112) | 112 | PC | 4 | 69.4 | 34 | 0 | NA | 26.9 | 16.6 |
| Bae 2000 | NA | mild to moderate AD | Korea | NA | 4 | N | 19 (19) | 19 | ITT | 4 | 69 | 37 | 0 | NA | 33.51 | 14.6 |
| Thal 1999 | NA | mild to moderate AD | USA | 24 | 24 | N | 117 (86) | 117 | ITT | 4 | 73.8 | 44 | NA | NA | 27.1 | 18.9 |
| Scharf 1999 | NA | mild to moderate AD | Australia | 1 | 25 | N | 17 (14) | 14 | ITT | 6 | 73.9 | 53.3 | NA | NA | NA | 17.79 |
| Rösler 1999 | NA | AD | Europe and North America | 45 | 26 | N | 239 (208) | 205 | PP | 6 | 72 | 41 | NA | NA | 23.29 | 19.9 |
| Jann 1999 | NA | mild to moderate AD | USA | NA | 6 | N | 133 (129) | 133 | ITT | 4 | 75 | 45.9 | NA | NA | 22.4 | 18.5 |
| Forette 1999 | NA | mild to moderate AD | International | 11 | 18 | N | 24 (22) | 19 | ITT | 4 | 72.5 | NA | NA | NA | 21.7 | 19.2 |
| Dubois 1999 | NA | mild to moderate AD | International | 71 | 26 | N | 208 (176) | 203 | ITT | 4 | 72 | 34.5 | 0 | NA | 24.48 | 18.68 |
| Burns 1999 | NA | mild to moderate AD | International | 82 | 24 | N | 274 (219) | 274 | ITT | 4 | 71 | 45 | NA | NA | NA | 20 |
| Álvarez 1999 | NA | mild to moderate AD | Spain | NA | 12 | N | 17 (NA) | 16 | NA | 3 | 73 | 35.3 | NA | 58.8 | 31.81 | 19.25 |
| Rogers 1998 | NA | mild to moderate AD | USA | 20 | 24 | N | 162 (130) | 153 | ITT | 5 | 72.6 | 39 | 0 | NA | 27.28 | 19.2 |
| Rogers 1998 | NA | mild to moderate AD | USA | 23 | 12 | N | 153 (0) | 150 | ITT | 4 | 74 | 39 | 0 | NA | 25.3 | 19.8 |
| Morris 1998 | NA | mild to moderate AD | USA | 24 | 26 | N | 135 (119) | 133 | ITT | 5 | 73.7 | 45.5 | 0 | NA | 20.4 | 19.4 |
| Imbimbo 1998 | NA | mild to moderate AD | USA and Italy | 26 | 25 | N | 106 (0) | 105 | ITT | 4 | 68.3 | 30 | NA | NA | 27.1 | 18.1 |
| Cummings 1998 | NA | mild to moderate AD | USA | NA | 12 | N | 120 (115) | 115 | ITT | 5 | 72 | 43 | 0 | NA | NA | NA |
| Weyer 1997 | NA | mild to moderate AD | Germany | 6 | 26 | N | 100 (0) | 100 | ITT | 5 | 70 | 31 | NA | NA | 23.3 | 18.1 |
| Rockwood 1997 | NA | mild to moderate AD | Canada | NA | 24 | N | 193 (0) | 187 | ITT | 4 | 71.5 | 47 | NA | NA | 20.5 | 19.6 |
| Bodick 1997 | NA | AD | USA and Canada | 17 | 24 | N | 87 (0) | 58 | PP | 4 | 75 | 43 | NA | NA | 23.0 | NA |
| Weyer 1996 | NA | mild to moderate AD | Germany | 14 | 52 | N | 153 (NA) | 153 | ITT | 4 | 68.9 | 35 | NA | NA | 34.3 | 16.8 |
| Shrotriya 1996 | NA | mild to moderate AD | USA | NA | 12 | N | 35 (32) | 35 | NA | 4 | 70.8 | 48.6 | 0 | NA | 16.2 | 22.5 |
| Rogers 1996 | NA | mild to moderate AD | USA | NA | 12 | N | 40 (35) | 40 | ITT | 5 | 70.6 | 47.5 | NA | NA | 27.2 | 18.2 |
| Huff 1996 | NA | mild to moderate AD | USA | NA | 12 | N | 91 (86) | 85 | PP | 4 | 71.7 | 49 | 0 | NA | 24.2 | 18.5 |
| Becker 1996 | NA | AD | USA | NA | 12 | N | 23 (23) | 23 | ITT | 4 | 72.3 | 39.1 | 0 | NA | 26.39 | 19.3 |
| Knapp 1994 | NA | mild to moderate AD | USA | 33 | 30 | N | 184 (128) | 116 | PP | 6 | 72.7 | 47 | 0 | NA | 29.2 | 18.2 |
| Farlow 1992 | NA | mild to moderate AD | USA and Canada | 23 | 12 | N | 158 (NA) | 158 | ITT | 4 | 71.3 | 45.9 | 0 | NA | 27.5 | 18.5 |

The Modified JADAD scale was used to assess the quality of trials, covering the aspects of randomization, concealment of allocation, double-blind, withdrawals, and dropouts.


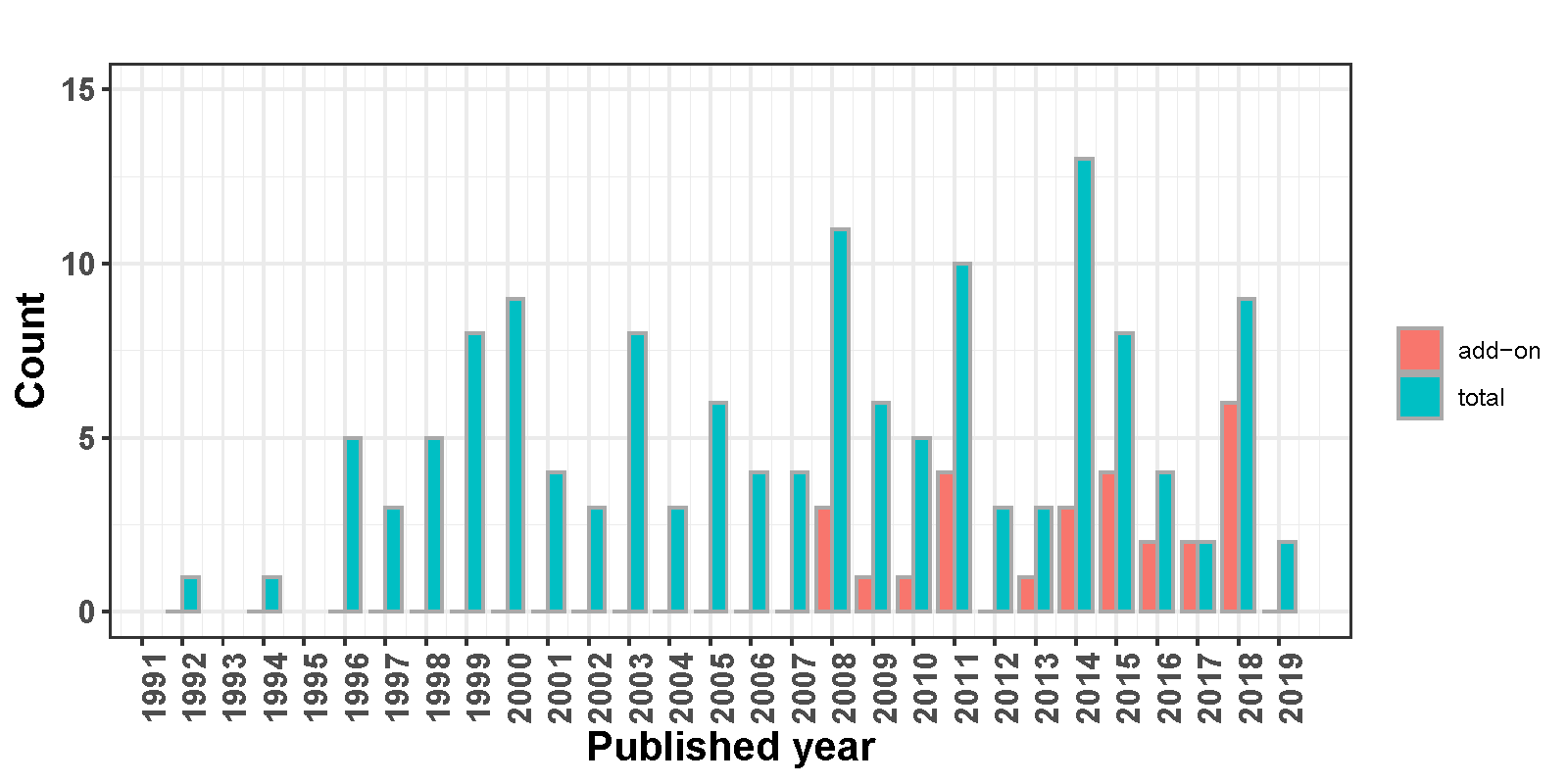


**Figure S1** Bar chart of the AD trials published from 1992 to 2019. The green bar represented the total number of publications for one year, the red bar represented some of them that utilized add-on design.


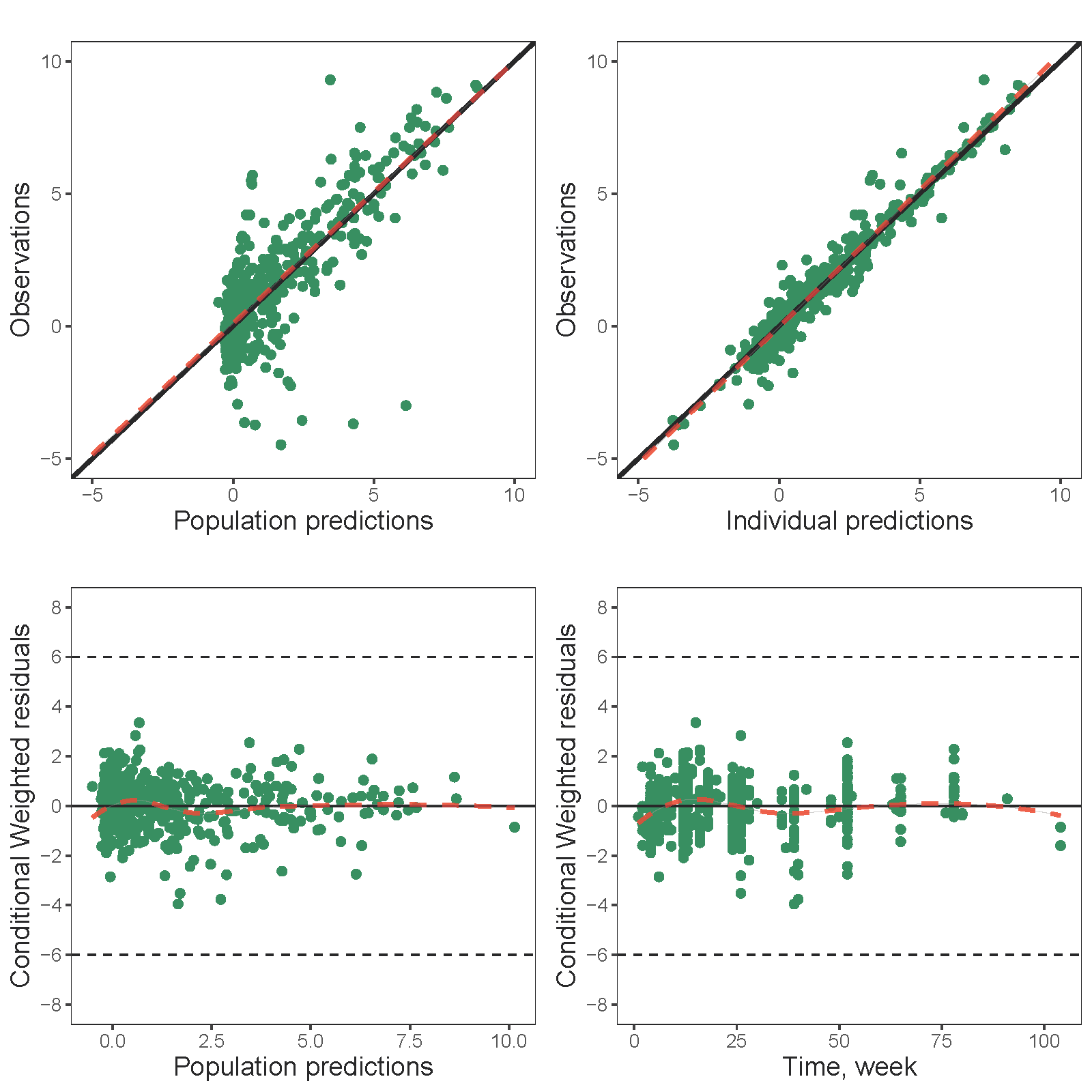


**Figure S2** Standard diagnostic plots of the final model. (A) Population predictions versus observations. (B) Individual predictions versus observations. (C) CWRESs versus population predicted value. (D) CWRESs versus time. The black solid line and red dotted line in (A) and (B) represent the identity line and linear regression line, respectively, whereas in (C) and (D) are the horizontal line where CWRES equal 0 and the red lines are the loess regression line.


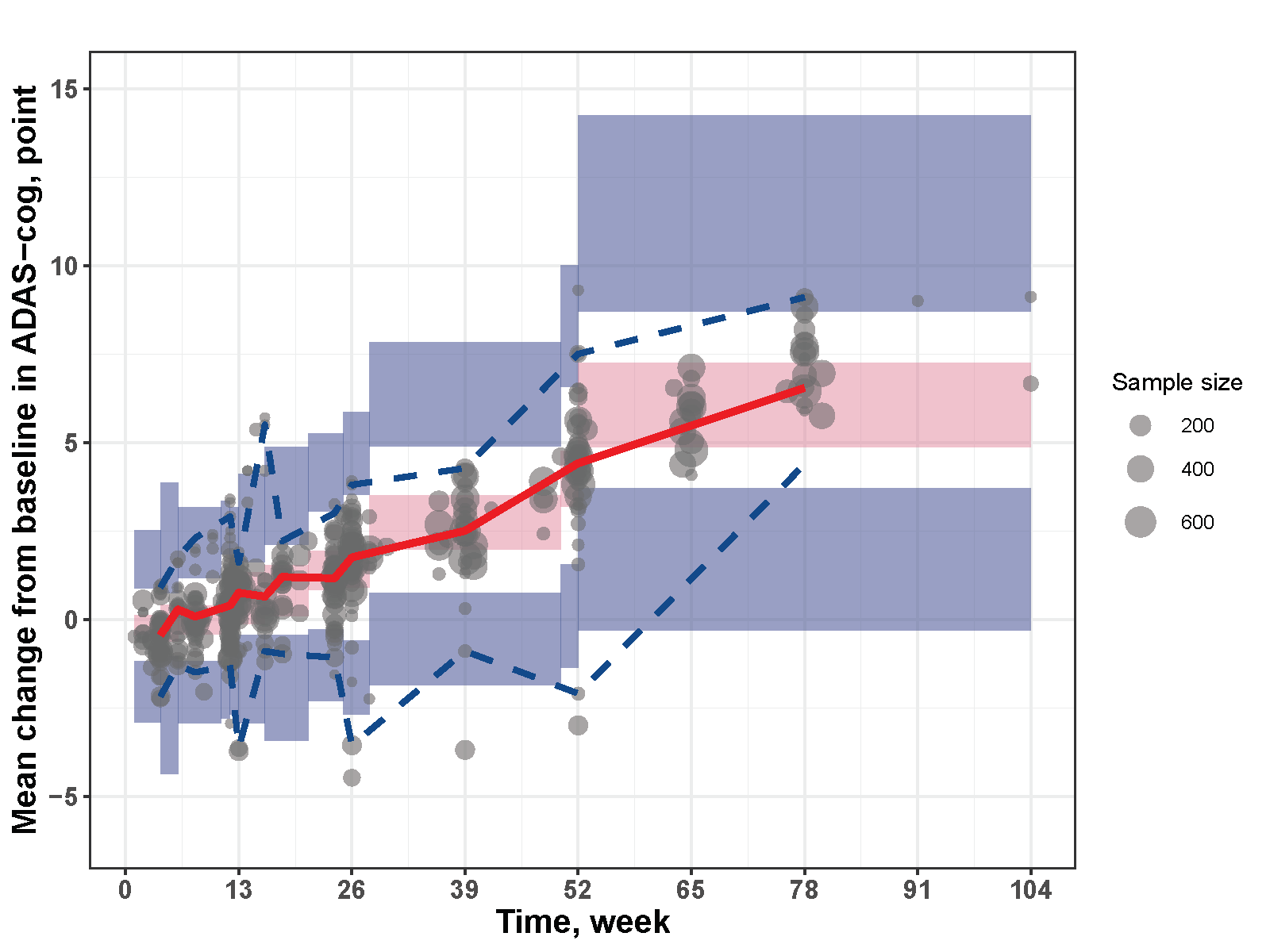


**Figure S3** Visual predictive check. The black points stand for the observed placebo response data and the point size is proportional to the corresponding sample size. The shadow areas are 95% confidence intervals for the 2.5th (blue), median (pink) and 97.5th (blue) percentiles. The red solid line is median value while the blue dotted lines are 2.5th and 97.5th percentiles based on the observed data.


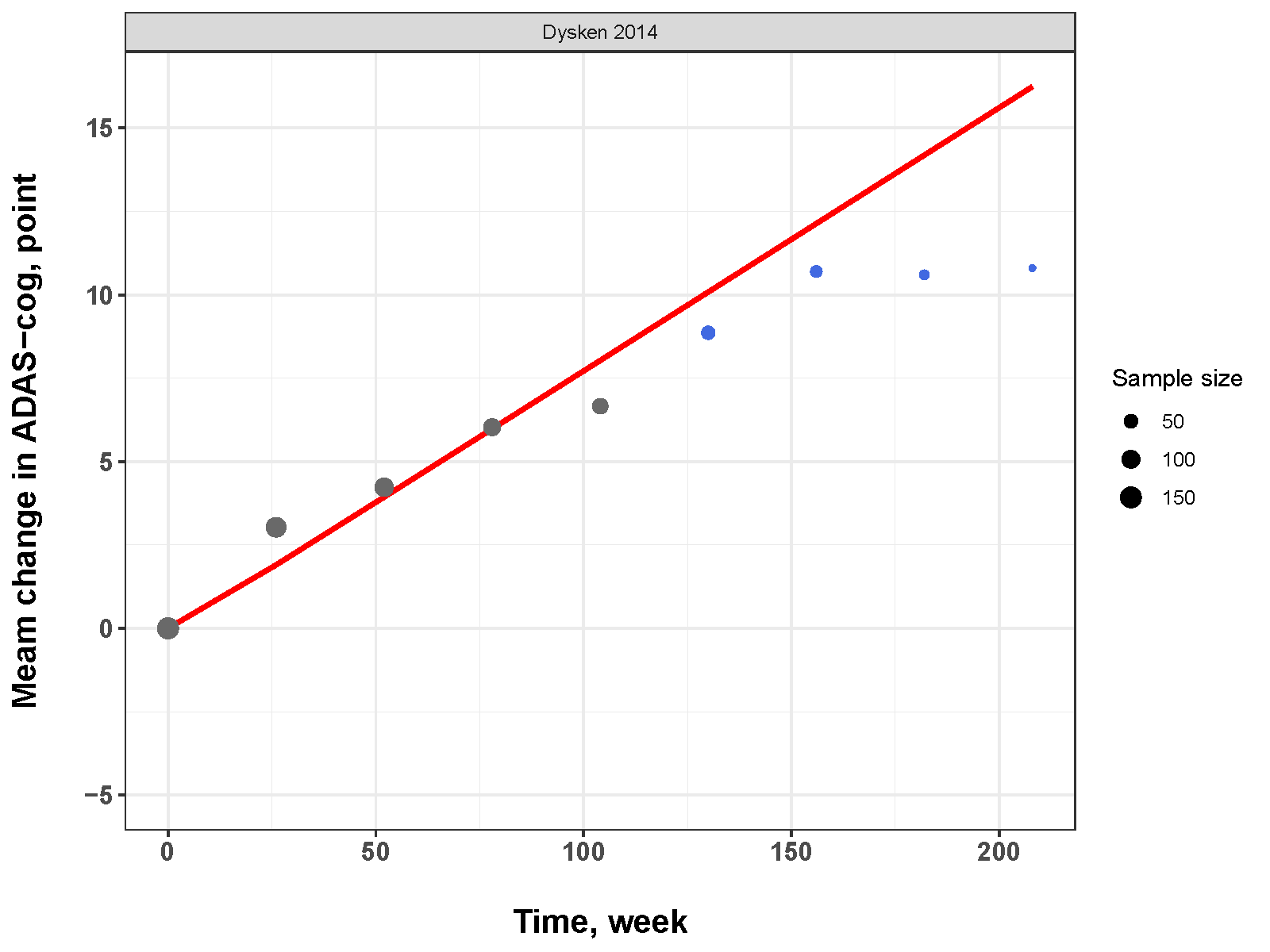


**Figure S4** Extrapolation of the final model prediction for the ADAS-cog change after two years. The long-term data for validation are extracted from two AD trials, Dysken et al. 2014 and Petersen 2005 et al. 2005. The black points are placebo data within two years utilized to establish the disease progression model, while the blue points are long-term data utilized for extrapolation validation. The point size is proportional to the corresponding sample size
